# Neuromotor Noise Is Malleable by Amplifying Perceived Errors

**DOI:** 10.1371/journal.pcbi.1005044

**Published:** 2016-08-04

**Authors:** Christopher J. Hasson, Zhaoran Zhang, Masaki O. Abe, Dagmar Sternad

**Affiliations:** 1 Department of Physical Therapy, Movement and Rehabilitation Sciences, Northeastern University, Boston, Massachusetts, United States of America; 2 Department of Bioengineering, Northeastern University, Boston, Massachusetts, United States of America; 3 Graduate School of Education, Hokkaido University, Sapporo, Hokkaido, Japan; 4 Departments of Biology, Electrical and Computer Engineering, and Physics, Northeastern University, Boston, Massachusetts, United States of America; 5 Center for the Interdisciplinary Research on Complex Systems, Northeastern University, Boston, Massachusetts, United States of America; Imperial College London, UNITED KINGDOM

## Abstract

Variability in motor performance results from the interplay of error correction and neuromotor noise. This study examined whether visual amplification of error, previously shown to improve performance, affects not only error correction, but also neuromotor noise, typically regarded as inaccessible to intervention. Seven groups of healthy individuals, with six participants in each group, practiced a virtual throwing task for three days until reaching a performance plateau. Over three more days of practice, six of the groups received different magnitudes of visual error amplification; three of these groups also had noise added. An additional control group was not subjected to any manipulations for all six practice days. The results showed that the control group did not improve further after the first three practice days, but the error amplification groups continued to decrease their error under the manipulations. Analysis of the temporal structure of participants’ corrective actions based on stochastic learning models revealed that these performance gains were attained by reducing neuromotor noise and, to a considerably lesser degree, by increasing the size of corrective actions. Based on these results, error amplification presents a promising intervention to improve motor function by decreasing neuromotor noise after performance has reached an asymptote. These results are relevant for patients with neurological disorders and the elderly. More fundamentally, these results suggest that neuromotor noise may be accessible to practice interventions.

## Author Summary

It is widely recognized that neuromotor noise limits human motor performance, generating errors and variability even in highly skilled performers. Arising from many spatiotemporal scales within the physiological system, the intrinsic noise component is commonly assumed to be invariant by most computational models of human neuromotor control. We challenge this assumption and show that after an individual has reached a performance plateau, amplifying perceived errors elicits continued reductions in observed variability. Model-based analyses show that the main driver of this effect is a reduction in the variance of neuromotor noise. Thus, error amplification has the potential to become a key intervention for individuals with increased movement variability due to high levels of neuromotor noise, ranging from children with dystonia, through patients with stroke, to healthy elders.

## Introduction

A hallmark of human movement is its variability, recognized in the phrase “repetition without repetition” [1]. Even the world champion in dart throwing does not hit the bull’s eye every time—in fact, it is this remaining randomness that creates the thrill in championships. This variability arises from interactions between a number of computational and physiological processes. Computationally, overt movement variability stems from imperfect error corrections and exploratory actions, and can sometimes be decreased by channeling variability into task-irrelevant dimensions [2–4]. Physiologically, some of the observed variability arises from a large number of processes at all levels of the neuromotor system, from noise in ion channel dynamics and action potential firing rates, to changes in the amounts of neuromodulators, such as serotonin or norepinephrine, that themselves depend on systemic factors, such as arousal [5–7]. While world-class champions may be close to a minimum in both computational and physiological sources of variability, older adults and patients with various neurological diseases show greater variability in their limb movements. For the latter, a behavioral intervention or therapy that decreases this overt variability and its underlying sources could have a major impact on motor function and quality of life. However, it is yet unclear whether or how this intrinsic noise can be accessed through behavioral interventions.

Any intervention targeting undesired variability in human movement must consider a fundamental component of motor performance: error. Error information is needed for learning and, consequently, manipulation of error influences learning [8, 9]. Error is typically manipulated physically or visually. Physically, external forces can guide an individual towards or away from a desired trajectory, causing either error reduction or amplification [10–15]. Visually, non-veridical action outcomes can be displayed that are better or worse than the actual performance. Importantly, studies on visual error amplification have shown that the manipulation accelerates learning and achieves enhanced performance [16–19].

Despite the promise of error amplification, the ways that this manipulation affects the human sensorimotor system remain unclear. Mechanistically, error amplification may influence how individuals correct for errors, i.e. changing the error correction gain (the proportion of error corrected on each trial). It has been proposed that error amplification may simply encourage an individual to make larger corrections, thus improving performance [18, 19]. While plausible, there may be more explanations for the observed benefits, which may not be apparent without a different approach. Prior work has used the framework of motor adaptation, where externally imposed errors are central and are gradually diminished to reinstate baseline performance. These previous studies have only assessed the effects of error augmentation on the exponential decline of errors, without separating this decay constant from the error correction gain. Further, the error correction gain may depend on the level of neuromotor noise, as the presence of noise may cause instability with a large gain [20].

To achieve a better understanding of the mechanisms underlying performance improvement with error amplification, computational learning models may be of significant value. Van Beers [21] used a stochastic state space model for a pointing task; the model introduced noise during two sequential stages of the error correction process. Modeling of the time course of this pointing task suggested that the optimal correction gain depended on the relative magnitude of the two noise sources. Using the same model to account for learning a throwing task, Abe and Sternad [22] showed that the error correction gain increased with practice. Hence, if one assumed that humans tend to use conservative gains below the optimal values to avoid instability, then it is reasonable to expect that error amplification improves performance by encouraging larger correction gains. Alternatively, the neuromotor system could lower its level of random noise, by changing computational strategies, such as channeling variability into task dimensions that reduce the error sensitivity of movement strategies [2, 3, 23, 24, 25]. Or, more simply, the system may lower the amplitude of its intrinsic noise via more system-level physiological mechanisms. The present study questions to what degree this is possible.

Physiological sources of noise are ubiquitous on all scales, from molecular to cellular to systemic. However, it is yet unknown whether they are modifiable by practice and interventions. This study explores whether error amplification could act as a simple aid to not only alter control strategies, but also lower neuromotor noise. A simple throwing task in a virtual environment was used as an experimental platform to test whether visual amplification of error affects motor performance, not only by changing how errors are corrected, but also, more importantly, by modifying neuromotor noise. Our focus was on improvements after performance has plateaued: by examining “already good” performance, the effects of error amplification are isolated from initial learning transients and sensorimotor calibration processes.

To further probe the status of this intrinsic noise, we not only used deterministic error amplification but also used stochastic amplification, where the amplification included a random component. We reasoned that layering additional noise on the amplified error would magnify any effects on intrinsic noise. Amplifying error deterministically makes performance appear worse by proportionally increasing both the magnitude and variability of errors, i.e. the coefficient of variation remains relatively unchanged. By disproportionately increasing variability by adding additional randomness, the coefficient of variation will increase. We conjectured that stochastic error amplification would make subjects perceive themselves as more noisy and variable compared to deterministic amplification alone, which would increase the pressure to reduce noise.

The experiment tested the following hypotheses: *Hypothesis 1*: Amplifying perceived errors improves task performance. *Hypothesis 2*: Adding noise to the amplified errors improves task performance more than deterministic error amplification alone. Three more hypotheses were tested using three stochastic iterative learning models (including an error correction gain and noise terms) to analyze the temporal structure of the task performance data: *Hypothesis 3*: Error amplification increases the size of error corrections, i.e. it increases the correction gain. *Hypothesis 4*: Error amplification reduces intrinsic neuromotor noise. *Hypothesis 5*: Stochastic amplification reduces intrinsic noise more than deterministic error amplification. Finally, we expected that there is an optimal error amplification magnitude that has the greatest effect on task performance and intrinsic noise.

## Methods

### Participants

Forty-two right-handed healthy individuals (22 male; 20 female; age: 24±5 years) participated in the experiment. All subjects received an explanation of the experimental task; however, they were not informed about any manipulations. Subjects read and signed an informed consent document approved by Northeastern University’s Institutional Review Board.

### Task

The task was designed to emulate the skill of throwing an object to hit a target. Specifically, the task was modeled after the table version of the “skittles” pub game (or tetherball). In this game, a ball is suspended from a string or chain attached to the top of a vertical post, and the ball is thrown to hit a target skittle on the other side of the post. The trajectory of the ball is fully determined by the ball’s release angle *θ*_*R*_ and angular velocity $\dot{\theta_{R}}$. The game is under-determined or redundant, as there are an infinite number of *θ*_*R*_ and $\dot{\theta_{R}}$ combinations that result in a successful hit for a given skittle location [23, 25].

### Experimental Setup and Data Acquisition

**Fig 1:**
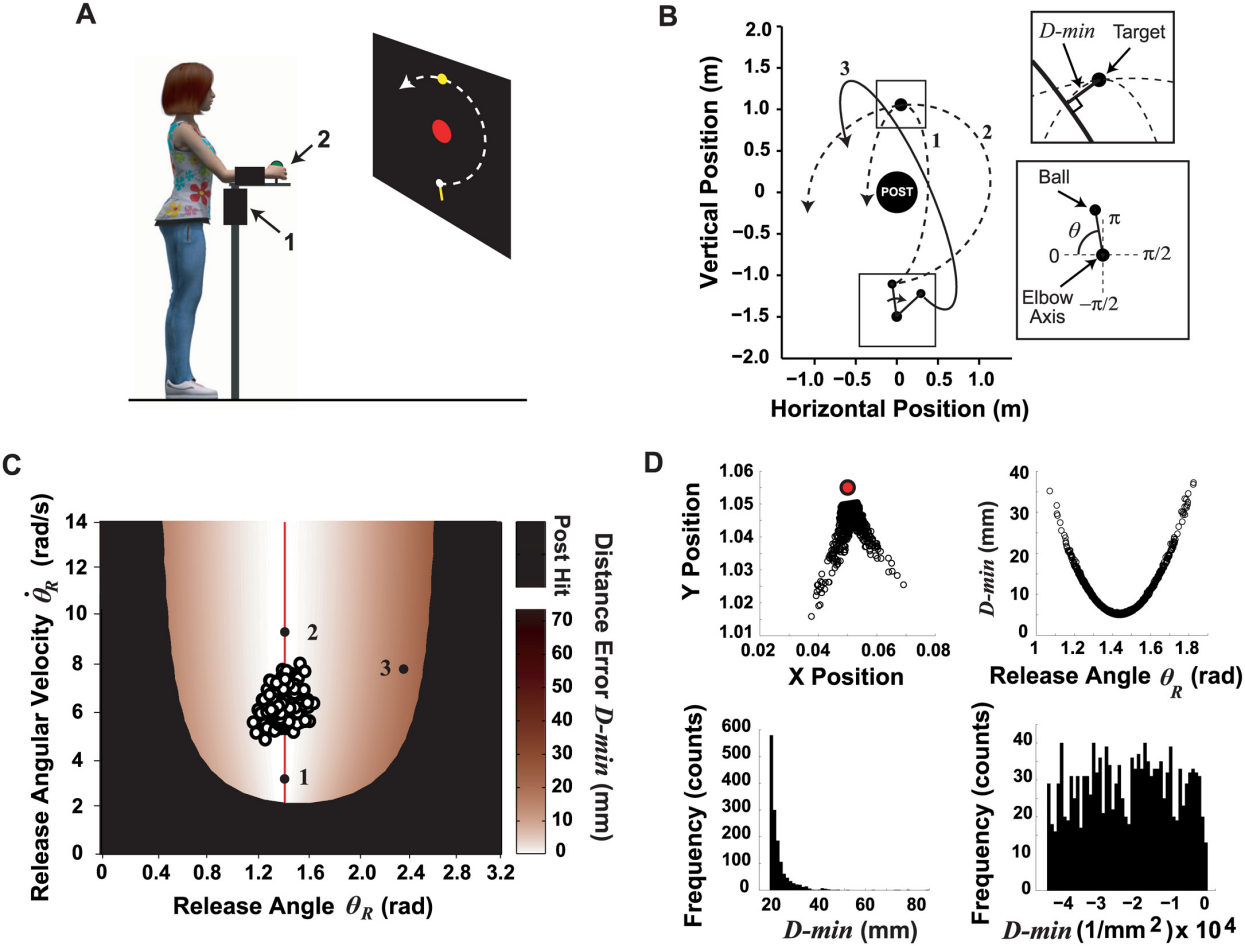
Experimental task. **A**: Schematic of the experimental setup for the skittles task; 1: potentiometer, 2: switch. The projector screen shows participant’s view of the virtual task: the center post is in the middle (red), the target skittle at the top (yellow), and the lever arm/ball is at the bottom (white). **B**: Schematic of the *x-y* workspace (top down view), illustrating three example trajectories. Trajectories 1 and 2 have the same release angle *θ*_*R*_, but different release velocities $\dot{\theta_{R}}$, and both score a perfect hit; trajectory 3 has a larger error. Insets show definitions of error *D-min* and the execution variable ball angle *θ*_*R*_. **C**: Execution and result space shows the error as a function of *θ*_*R*_ and $\dot{\theta_{R}}$. The solution manifold is shown by the red line, corresponding to *θ*_*R*_ and $\dot{\theta_{R}}$ combinations that result in the smallest error (*D-min*). An example of 240 throws produced by an experienced skittles player is shown (hollow circles), as well as the three artificial numbered data points showing different task executions (corresponding to the three trajectories shown in panel B). The solution manifold for the chosen target location was insensitive to velocity, as can also be seen from ball releases 1 and 2, which have different release velocities but both score direct hits. **D:** Points at which the ball came closest to the target, i.e. *D-min* (in the x-y workspace), visualized for one exemplar subject (upper left sub-panel). The relation between *θ*_*R*_ and *D-min* is nonlinear (upper right sub-panel), also showing the redundancy: any given error can be achieved with more than one release angle. The distribution of *D-min* has an extreme positive skew (lower left sub-panel). Applying the transformation -1/*D-min*^2^ makes the distribution more symmetrical to permit calculation of an average value (lower right sub-panel).

Participants practiced a two-dimensional version of the skittles throwing task in a virtual set-up with an instrumented lever arm that rotated in the horizontal plane (Fig 1A). A cushioned splint was fixed to the lever arm to secure the participant’s dominant arm in the apparatus. A wooden ball of 6 cm diameter was fixed to the end of the lever arm and subjects grasped this ball with their hand. The distance between the lever arm axis of rotation and the ball was adjustable to the subject’s arm length. The angular position of the skittles lever arm *θ* was measured with a potentiometer (Bourns, Inc., Riverside, CA). A force-sensing resistor (Trossen Robotics, Westchester, IL) positioned on the surface of the ball was used as a switch to indicate release by converting the analog force signal to a binary open/closed signal using a Schmidt Trigger (Fairchild Semiconductor Corp., South Portland, ME). The switch was positioned so that the index finger closed the switch when the ball was grasped. The switch opened when the finger was extended and this simulated ball release. Data were sampled at 700 Hz using a personal computer and 16-bit analog-to-digital converter (DT300, Data Translation, Inc., Marlboro, MA). A rear-projection screen (width: 1.8 m; height: 1.4 m) was placed 0.6 m in front of the subject and the skittles manipulandum.

A custom-written acquisition program (Visual C++, Version 6.0, Microsoft, WA) sampled *θ*_*R*_ and the switch state and gave visual feedback to participants (Open GL, Version 1.2, Silicon Graphics, CA). Angular velocity $\dot{\theta_{R}}$ was calculated online using a linear regression over the most recent 10 data samples. At ball release, the slope of the regression was used as the velocity estimate. (Regression attenuated the influence of measurement noise.) During task performance the positions of the lever arm, ball, center post, and target skittle were displayed on the rear-projection screen (Fig 1A). The participants saw a top-down projection of the workspace; there were no reports of difficulty with this 90° transformation. When the lever arm was moved and the virtual ball was released (at the switch opening time), the program calculated the ball’s trajectory using the model of Müller and Sternad [23]:

$$x\left(t\right)=A_{x}\text{sin}\left(\omega t+\varphi_{x}\right)e^{-\left(\frac{t}{\tau }\right)}$$ (1)

$$y\left(t\right)=A_{y}\text{sin}\left(\omega t+\varphi_{y}\right)e^{-\left(\frac{t}{\tau }\right)}$$ (2)

where *τ* was the time constant of the decaying trajectory (*τ* = 20 s), with amplitudes *A*_*x*_ and *A*_*y*_, given by

$$A_{x}=\sqrt{x_{R}^{2}+{\left[\frac{{\dot{x}}_{R}}{\omega }+\frac{\left(\frac{x_{R}}{\tau }\right)}{\omega }\right]}^{2}}$$ (3)

$$A_{y}=\sqrt{y_{R}^{2}+{\left[\frac{{\dot{y}}_{R}}{\omega }+\frac{\left(\frac{y_{R}}{\tau }\right)}{\omega }\right]}^{2}}$$ (4)

where *x_R_*, *y_R_* and ${\dot{x}}_{R}$, ${\dot{y}}_{R}$ are the positions and velocities of the ball at release, respectively, with *E*_*x*_ and *E*_*y*_ denoting the kinetic and spring potential energies determined by

$$E_{x}=0.5\left(m{\dot{x}}_{R}^{2}+kx_{R}^{2}\right)$$ (5)

$$E_{y}=0.5\left(m{\dot{y}}_{R}^{2}+ky_{R}^{2}\right)$$ (6)

where *m* = 0.1 kg and *k* = 1.0 N/m. The phases *φ*_*x*_ and *φ*_*y*_ of the sinusoidal motions of the two springs were based on the oscillation amplitudes and the *x* and *y* release positions (*x*_*R*_ and *y*_*R*_, respectively) and are given by

$$\varphi_{x}=arccos\left[\frac{1}{A_{x}}\left(\frac{{\dot{x}}_{R}}{\omega }+\frac{\left(\frac{x_{R}}{\tau }\right)}{\omega }\right)\right]$$ (7)

$$\varphi_{y}=arccos\left[\frac{1}{A_{y}}\left(\frac{{\dot{y}}_{R}}{\omega }+\frac{\left(\frac{y_{R}}{\tau }\right)}{\omega }\right)\right]$$ (8)

and the natural frequency *ω* was

$$\omega =\sqrt{\frac{k}{m}-\frac{1}{\tau^{2}}}=3.16\;\text{rad/s}$$ (9)

After the ball passed the target skittle, the minimum distance between the ball trajectory and center of the target skittle *D-min* was calculated (Fig 1B). The color of the target skittle changed from yellow to red, if *D-min* < 0.011 m, signaling a successful hit to the subject.

### Task Workspace and Solution Space

The post (radius = 0.25 m) was in the center of the workspace at *x* = 0, *y* = 0 m, while the elbow axis of rotation was located at *x* = 0, *y* = -1.5 m. The target skittle (radius = 0.05 m) was positioned at *x* = 0.05, *y* = 1.05 m. Fig 1B illustrates three different trajectories and the two insets show the coordinates of the arm including the target and error definition. Fig 1C shows the execution space that represents all possible combinations of the execution variables *θ*_*R*_ and $\dot{\theta_{R}}$ and the result variable *D-min*. Combinations of ball release angle and velocity, *θ*_*R*_ and $\dot{\theta_{R}}$, that give exact target hits, *D-min* = 0, define the solution manifold, which is shown by the red vertical line. For this study, the target position was chosen such that errors depended primarily on *θ*_*R*_ and only very little on $\dot{\theta_{R}}$, i.e. the solutions were largely insensitive to release velocity. The release angle that yielded a perfect hit was *θ*_*Target*_ = 1.44 rad. An example of 240 throws produced by an experienced skittles player is shown in the solution manifold, as well as three artificial numbered data points showing different possibilities. Note that other target constellations are associated with nonlinear U-shaped solution manifolds where the *D-min* depends on both release angle and velocity, *θ*_*R*_ and $\dot{\theta_{R}}$ [25].

Specific to this target constellation was also that the ball could only pass below the target and the smallest achievable distance error (*D-min*) was 5 mm, corresponding to *θ*_*R*_ = 1.44 rad. In Fig 1D, the top left panel illustrates the *x-y* workspace with the target in red; each black circle represents closest approach of the ball (*D-min*) for a set of exemplary trajectories. Note that subjects could distinguish between the deviations to the left and the right branch from the perfect release angle by the elliptic shape of the ball trajectories. Therefore, error corrections based on visual information were relatively straightforward. The top right panel of Fig 1D shows that the relation between *θ*_*R*_ and *D-min* is nonlinear and approximately parabolic.

Given the geometry of the workspace and the definition of error *D-min* as always positive, the errors typically assumed a very skewed distribution (Fig 1D, lower left panel). For statistical analysis, we therefore transformed this data using *−*1*/x*^2^, based on Tukey's "ladder of powers" [26]. This choice was made after trying several transformations of increasing severity until an approximately uniform distribution was obtained that afforded calculating means (Fig 1D, lower right panel). For the presentation of the results, the errors were transformed back.

### Experimental Manipulation

To manipulate the distance error *D-min*, only the release angle *θ*_*R*_ had to be modified, as the solution manifold was insensitive to variations in the release velocity $\dot{\theta_{R}}$. Therefore, the release angle was measured online to calculate the observed ball trajectory and then modified to amplify the error as follows

$${\tilde{\theta }}_{R}=\theta_{Target}+A\left(\theta_{R}-\theta_{Target}\right)$$ (10)

where ${\tilde{\theta }}_{R}$ denotes the manipulated release angle, *θ*_*Target*_ the release angle that gave a perfect hit, i.e. *D-min* = 0, and *A* the amplification gain. Here, the magnitude of error amplification is held constant (for a given *A*), and therefore this manipulation is called deterministic error amplification, or DEA. Three magnitudes of *A* were applied: *A* = 1.5 (DEA-1.5), *A* = 2.0 (DEA-2.0), and *A* = 2.5 (DEA-2.5). Note that the ball trajectory that the participants saw was calculated based on ${\tilde{\theta }}_{R}$, while the angular velocity $\dot{\theta_{R}}$ remained unchanged.

To stochastically amplify error (SEA), the manipulation was

$${\tilde{\theta }}_{R}=\theta_{Target}+\left(A+\xi \right)\left(\theta_{R}-\theta_{Target}\right)$$ (11)

where ξ was uniformly distributed noise on the interval [−(*A*−1), +(*A−*1)]. These interval boundaries ensured that the noise was centered on *A* and scaled with *A*. For example, for *A* = 2.0 the noise interval was [–1, 1] creating random amplification gains in the range of [1, 3]. For *A* = 1.5 the range was [1, 2], for *A* = 2.5 the range was [1, 4]. Corresponding to the DEA levels, the three mean or effective gains $\bar{A}=A+\xi$ were: $\bar{A}=1.5$ (SEA-1.5), $\bar{A}=2.0$ (SEA-2.0), and $\bar{A}=2.5$ (SEA-2.5). Errors were only amplified up to a limit; if the manipulated release angle ${\tilde{\theta }}_{R}$ fell outside the range of 1.00 to 1.88 rad (optimum = 1.44 rad) subjects saw their true error. This prevented subjects from noticing the manipulations. Pilot work had shown that when subjects perceived very large errors, they “discount” them as external perturbations [27].

### Experimental Protocol

**Fig 2:**
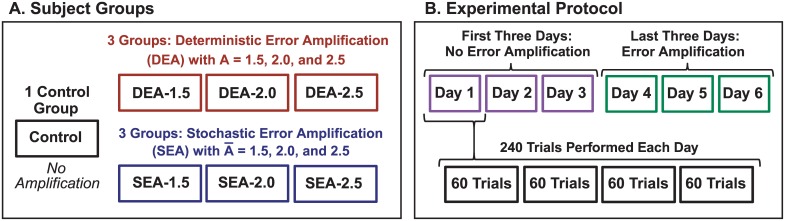
Experimental design and protocol. Three subject groups received deterministic error amplification (DEA) of varying amplitudes *A* with a gain of 1.5, 2.0, and 2.5. Three other groups received stochastic error amplification (SEA), in which a sample from a uniform white noise was added to the amplified error (with mean amplification factors *A* of 1.5, 2.0, and 2.5). A control group received no error manipulation. All groups practiced the task for three days without manipulations; on three subsequent days, the six experimental groups, DEA and SEA, received error amplification.

The 42 subjects were randomly assigned to one of seven groups: there were six experimental groups that received varying types and amounts of error amplification, and one control group without manipulation (Fig 2). Three groups received DEA and three received SEA. Each amplification type was applied at three levels: 1.5, 2.0, and 2.5. The experiment comprised 6 daily sessions of practice divided into two stages: On the first three days of practice, all participants performed the virtual task without any experimental manipulations. During the second three days, the visual feedback was altered for the six experimental groups (DEA and SEA). Altogether, there were 252 separate practice days with data collection. On each practice day the participants performed four blocks of 60 trials each, to yield 240 trials per day and 1440 total trials for each subject.

### Procedure

Participants placed their right forearm into the cushioned splint attached to the lever arm. The height of the lever arm and the distance between the axis of rotation and the ball were adjusted to place the forearm at a comfortable height with the ball firmly grasped. The participants were instructed to hit the center of the target skittle with the ball and to avoid hitting the center post. Each throw typically lasted 1–2 s with about 3 s between the self-initiated throws. The experiment duration in each daily session was about 20 min.

### Data Reduction

For each subject/day the 240 trials were parsed into non-overlapping bins of 20 trials. Participants occasionally hit the center post; these trials were replaced using bootstrap resampling [28]. This method computed an estimate of the sampling distribution of the non-post-hit trials and then replaced the post-hit with a new value randomly drawn from the estimated distribution. Trials with post hits were not eliminated because this would have disrupted the iterative trial-to-trial process, which was the basis for the second set of analyses on the temporal structure of the data using system identification. Within each 20-trial bin the data were averaged to obtain the mean distance error *D-min*. While the -1/*x*^2^ transformed *D-min* was used for statistical analysis, the figures display the more intuitive untransformed *D-min* values. A preliminary analysis of *D-min* for Day 3 revealed one outlier subject in the group DEA-2.0 and one subject in the group SEA-2.5, whose means were outside 1.5 times the interquartile range (±2.7 standard deviations or 99.3% of a normal distribution). These two subjects were excluded from the analysis; thus, for DEA-2.0 and SEA-2.5 the number of participants was five. Note that the variations in *θ*_*R*_ were not analyzed, because after initial practice these variables were centered on the optimal release angle and therefore showed the same patterns as the mean *D-min*. This is consistent with previous work that showed that data distributions centered on the most error-tolerant location early in practice [2].

### Statistical Analysis

Due to the extensive experimental protocol, i.e. seven groups with six days of practice for each subject, the number of subjects in each group was relatively small and did not satisfy the assumptions of ANOVA: normality and equality of variances. Therefore, the data were analyzed with permutation tests, which are a subset of non-parametric statistics [29, 30]. This analysis method uses permutations to create a sample-specific distribution, instead of using an assumed theoretical distribution. A cut-off for a given *p*-value was obtained from the specific distribution. The permutation analysis involved several steps. First, the data from all subject groups were pooled, based on the null hypothesis that all groups were part of the same population. In the subsequent resampling procedure, the data were randomly shuffled, split into two groups, and the difference between the group means was recorded. This procedure was repeated 1,000,000 times, resulting in a distribution of group mean differences that represented the probability of obtaining a given difference between two groups randomly selected from the subject population.

For all statistical comparisons the difference between the relevant means was compared with the bootstrap distribution. The *p*-value for each comparison was calculated by dividing the number of bootstrap differences smaller than the actual group difference by 1,000,000 and multiplying by 2 (to give the *p*-value for a two-tailed test). The critical threshold for significance was set to *p* < .05, meaning that there had to be 25,000 or fewer bootstrap differences below the actual group difference. For example, if only 1000 bootstrap differences were below the tested group difference, the *p*-value for a two-tailed test was *p* = .002.

To evaluate *Hypothesis 1*—amplifying perceived errors improves task performance—statistical tests were performed to test for differences in *D-min* between each error amplification group and the control group. These tests were conducted for Day 3, Day 6, and the change or difference between Day 3 and 6. Evaluating *D-min* on Day 3 tested for between-group baseline differences before error amplification was applied. Evaluating *D-min* on Day 6 assessed the level reached after three days of practice with amplified error. Examining the change between the days (Day 6 –Day 3) provides a direct assessment of the manipulation effects over time. To test *Hypothesis 2*—stochastic amplification of perceived errors improves task performance more than deterministic error amplification—the *D-min* for each pair of DEA and SEA groups (within an error amplification level, i.e. 1.5, 2.0, or 2.5) was compared. Finally, to test for possible differences among error amplification levels, i.e. is there an optimal error amplification gain, each DEA group was compared with each other, and each SEA group was compared with each other. The transformed *D-min* (−1*/x*^2^) was used for all statistical tests.

### System Identification Methods

To test *Hypotheses 3*, *4*, and *5*, which were concerned with the effects of error amplification on the error correction gain and neuromotor noise, we analyzed the error time series across practice using system identification techniques with three different learning models. Results of system identification are evidently dependent on the model used. Therefore, we tested three models that presented a step-wise increase in complexity. All three models included the basic component of any learning model—an error correction gain *B*. Two of the models quantified intrinsic motor noise via a single noise source, and the third model introduced two independent noise sources. Due to the different model structures they required different methods of system identification. The goal of these analyses was to tease apart contributions in the overt performance change due to the error correction gain or from noise. Note that results revealed that Model 1 and 2 were not as suitable as Model 3. We nevertheless present all three models to highlight that the model structure significantly determined the results. However, as the data show, one result was invariant across the three model structures. Details about the models and procedures follow.

### Model 1

The first model contained two iterative steps with the addition of one noise sample, described by the following equations [21]:

$$\theta_{R,i}=\theta_{PL,i}+\eta_{i}$$ (12)

$$e_{i}=\theta_{R,i}-\theta_{Target}$$ (13)

$$\theta_{PL,i+1}=\theta_{PL,i}-Be_{i}$$ (14)

where *θ*_*PL*,*i*_ is the planned release angle at trial *i*, *B* is the error correction gain, *θ*_*R*,*i*_ is the actual release angle, *η*_i_ is a sample from a zero-mean Gaussian distribution with variance equal to *σ*^2^, *e*_*i*_ is the error between the release angle *θ*_*R*,*i*_ and the angle that hits the target *θ*_*Target*_. This model assumes that the actual executed release angle *θ*_*R*,*i*_ is equal to the internally planned release angle *θ*_*PL*,*i*_ with added motor noise *η*_i_ (the labels “planning” and “execution” should not be taken literally). The planned angle *θ*_*PL*,*i*_ is updated trial-by-trial according to the visual error and correction gain *B*.

Note that either the actual or manipulated release angle, *θ*_*R*_ or ${\tilde{\theta }}_{R}$, could be used in the system identification. The interpretation of *B* was dependent on this choice. Using *θ*_*R*_ means that if participants fully adjusted the size of their corrections in response to error amplification, then an increase in *B* should be observed, and this increase should match the error amplification gain. If ${\tilde{\theta }}_{R}$ was used, then *B* remained unchanged if participants increased the size of their corrections in proportion to the amplified errors. For modeling SEA effects, using an amplified ${\tilde{\theta }}_{R}$ is non-trivial, because an additional noise term would be needed. To minimize the number of unknown model parameters we used *θ*_*R*_ in the system identification.

To estimate the two unknown parameters *B* and *σ*^2^, the equations were rearranged and combined into a single equation; *θ*_*Target*_ was set to zero. First, Eq 12 was increased by one iteration step:

$$\theta_{R,i+1}=\theta_{PL,i+1}+\eta_{i+1}.$$ (15)

Then, Eqs 12 and 15 were inserted into Eq 14, giving

$$e_{i+1}=e_{i}\left(1-B\right)+\eta_{i+1}-\eta_{i}.$$ (16)

System identification was applied to the time series of execution angles *θ* with the target angle subtracted, according to Eq 13. Model validation procedures showed that the system identification of Model 1 was associated with positive biases in *σ*^2^ (see S1 Appendix). In addition, system identification of the experimental data showed that estimates for *B* were close to zero, and prior research has shown that modeling with only one noise source was inadequate [21, 22]. Thus, we introduced Model 2 that added a second source of noise.

### Model 2

This model is a simple extension of Model 1 by adding a second sample of motor noise into the “planning” stage:

$$\theta_{R,i}=\theta_{PL,i}+\eta_{EX,i}$$ (17)

$$\theta_{PL,i+1}=\theta_{PL,i}-Be_{i}+\eta_{PL,i+1}$$ (18)

$$K=\frac{\eta_{EX,i}}{\eta_{EX,i}+\eta_{PL,i}}=\frac{\eta_{EX,i}}{\eta_{TOTAL,i}}$$ (19)

where *η*_*TOTAL*,*i*_ was a sample drawn from a zero-mean Gaussian distribution with variances equal to *σ*^2^. *η*_*TOTAL*,*i*_ was separated into *η*_PL_ and *η*_EX_ and their magnitudes were constrained by the ratio *K*. With this constraint, *η*_PL_ and *η*_EX_ were not independent. The parameter *K* was used to describe the noise ratio (Eq 19). *B* was the error correction gain; the error in the release angle was given by:

$$e_{i}=\theta_{R,i}-\theta_{T\text{arg}et}.$$ (20)

To estimate the three unknown parameters *B*, *K*, and *σ*^2^ Eqs 17 to 20 were rearranged into the form of a regression equation as for Model 1:

$$e_{i+1}=e_{i}\left(1-B\right)+\eta_{TOTAL,i+1}-K\eta_{TOTAL,i}.$$ (21)

As can be seen, Model 1 is a particular case of Model 2 with *K* = 1. Again, system identification was performed on the angle errors as defined in Eq 21. The constraint *K* enabled system identification with a linear regression model. More importantly, this constraint “colored” the noise, i.e. the output noise showed long-range correlations with a 1/f distribution that depended on *K*. A wide range of studies ranging from brain oscillations to motor behavior, such as tapping, posture and walking, have shown that motor noise is colored [31–34]. In contrast to Model 1, validation of Model 2 showed reliable noise estimates (S1 Appendix). However, simulation results also showed that about 38% of the experimental data rendered negative *K* values (S2 Appendix). Based on the definition in Eq 19, *K* should be positive within [0, 1]. These results suggested that Model 2 was not appropriate for those blocks of data. Thus, we introduced Model 3 that separated execution and planning noise into two independent quantities.

### Model 3

In this model, we assumed that execution and planning noise were independently generated from two random processes. This model was previously shown to account for the observed structure in the variability of human motor actions [21, 22]. The equations describing the model’s behavior were:

$$\theta_{R,i}=\theta_{PL,i}+\eta_{EX,i}$$ (22)

$$\theta_{PL,i+1}=\theta_{PL,i}-Be_{i}+\eta_{PL,i+1}$$ (23)

$$e_{i}=\theta_{R,i}-\theta_{T\text{arg}et}.$$ (24)

where *η*_*EX*_ and *η*_*PL*_ were random samples from two independent zero-mean Gaussian noises with different noise variances; *e*_*i*_ denoted the angle error of sample *i*, and *B* was the error correction gain.

### Parameter Estimation

System identification was applied on the experimental data using Models 1, 2, and 3. Consistent with the models, the measured release angle *θ*_*R*_ was converted to error by subtracting the target angle (1.44 rad) from each data point. Note that the error in angle could be zero, unlike the distance error calculated in the x-y workspace. For each subject, the estimations were conducted for each block of 60 trials, yielding four separate estimates per day for each subject. Initial transients were eliminated by excluding the first 10 trials of Block 1 on each day.

For Models 1 and 2 the MATLAB System Identification Toolbox (version 9.2) was used with the function *pem*.*m* (Prediction Error Method) to find estimates for the unknown parameters (for Model 1: *B* and *σ*^2^; for Model 2: *B*, *K*, and *σ*^2^). This algorithm estimated the parameter vector Θ by minimizing the squared prediction error [35]:

$$\hat{\Theta }=\text{arg }{\text{min}}_{\Theta }\frac{1}{t}\Sigma_{i=1}^{t}\varepsilon_{i}^{2}\left(\Theta \right)$$ (25)

where *ε*_*i*_(Θ) = *θ*_*R*,*i*_ − *f*_*i|i−*1_(Θ) is the prediction error with Θ = {*K*,*B*,*σ*^2^} and *f*_*i*|*i*−1_(Θ) is an optimal predictor:

$$f_{i|i-1}\left(\Theta \right)=\left(1-B\right)e_{i}-K\eta_{i-1}$$ (26)

To optimize Eq 25, we applied a nonlinear least-square curve fitting algorithm with the Levenberg-Marquardt Method using the function lsqnonlin.m of the MATLAB Optimization Toolbox (version 7.2). Since Model 3 included two independent noise sources, a different identification method was needed. Thus, a Maximum Likelihood Estimation (MLE) was performed using the Expectation-Maximization (EM) algorithm [36] to identify *B*, *σ*_*PL*_^2^, and *σ*_*EX*_^2^. The maximum likelihood estimator of Eqs 22 and 23 was given by:

$$\hat{\Theta }=\text{arg }\underset{\Theta }{\text{max}}\text{ log}p\left(\theta_{R,1},\cdots ,\theta_{R,t}|\Theta ;\;e_{1},\cdots ,e_{t}\right)$$ (27)

where

$$\Theta \equiv \left\{-B,\sigma_{EX}^{2},\sigma_{PL}^{2}\right\}.$$ (28)

### Model Validation

To test the validity of the system identification methods, Monte-Carlo simulations with known parameters were performed for all three models, followed by the identification of the parameters. Details and results of the validation are shown in S1 Appendix. For Models 1 and 2, the estimation of *B* and *σ*^2^ had a significant positive bias; *K* was underestimated in Model 2, especially for higher values. In these cases, we used the Adjusted Yule-Walker (AYW) method [37] to provide an unbiased estimate of *B* (see S1 Appendix for details). On the other hand, validation of Model 3 showed that the estimation provided unbiased estimates of all model parameters.

### Data Reduction and Statistical Analyses

Based on the model validation and estimation results, Model 3 was deemed to be most appropriate. Therefore, only the results for this model are presented below and the results for Models 1 and 2 are relegated to S2 Appendix. Focusing on Model 3, three dependent variables were analyzed: the error correction gain *B*, the execution noise variance *σ*_*EX*_^2^, and the variance of planning noise *σ*_*PL*_^2^. Each parameter estimate was computed for Day 3, Day 6, and the change across the manipulation (Day 6–3). Hence, these measures received the same statistical treatment as the behavioral measures.

To test *Hypothesis* 3—error amplification increases the size of corrective actions—comparisons were made for *B* between each experimental group and the control group for Day 3, Day 6, and the change from Day 3 to Day 6. To test *Hypothesis* 4—error amplification reduces intrinsic neuromotor noise—similar comparisons were made for the two noise estimates. To test *Hypothesis 5*—stochastic amplification reduces noise more than deterministic error amplification—comparisons were made between DEA and SEA as already described for *D-min* above. The same tests were also performed to identify differential effects across the different amplification gains.

## Results

### Effects of Error Amplification on Task Performance (Hypotheses 1 & 2)

#### Mean distance error

**Fig 3:**
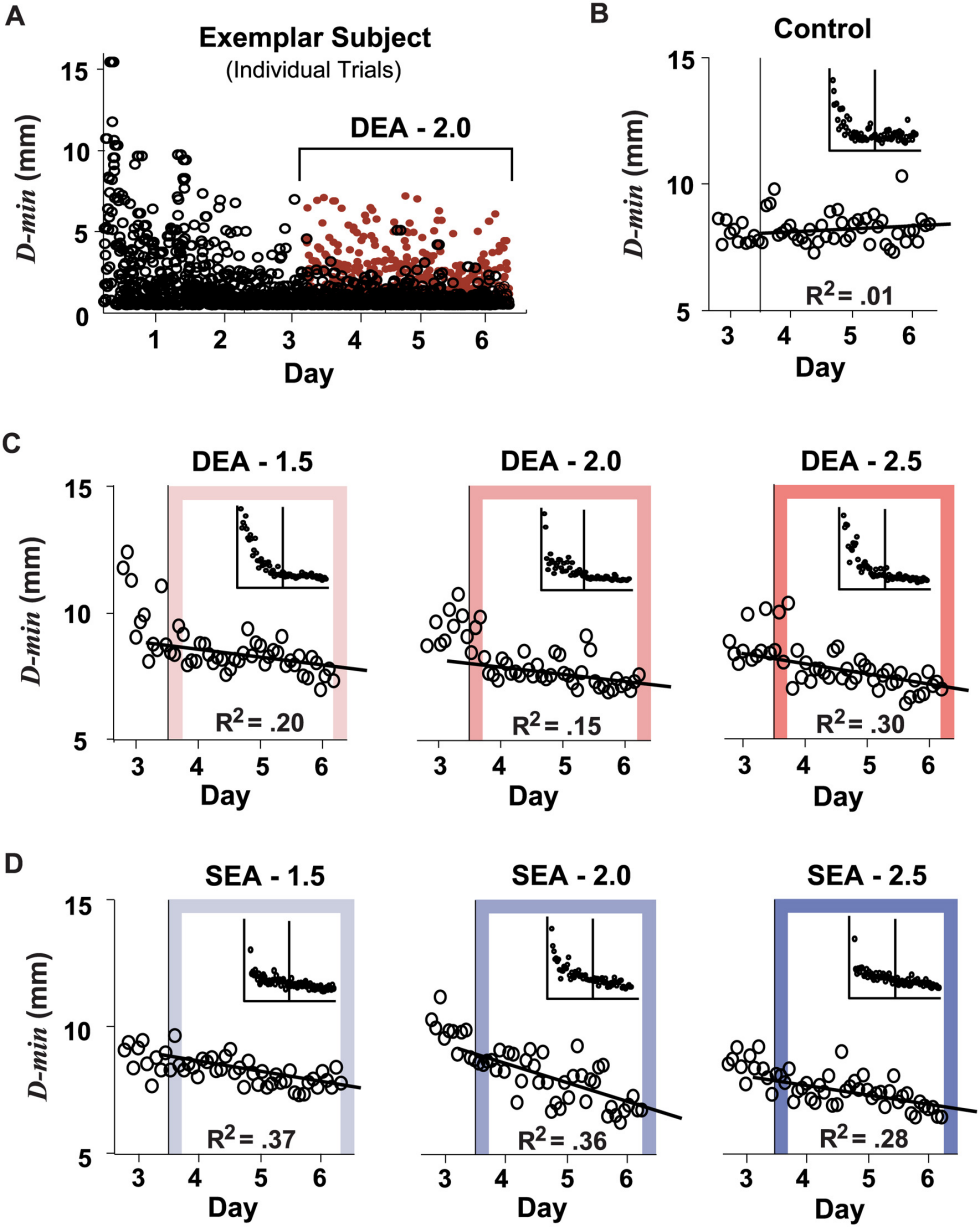
Minimum distance error *D-min* continually decreased in groups receiving error amplification. Panel **A** shows the raw *D-min* for an exemplar subject in the DEA-2.0 group; black hollow circles are the actual errors; the red circles are the amplified errors. Panels **B** to **D** show *D-min* for the last day of practice with no manipulation (Day 3) and three subsequent days (Day 4–6) in which subjects received either no error amplification (controls; panel **B**) or different magnitudes of deterministic or stochastic error amplification (DEA or SEA, respectively; panels **C** and **D**). For panels **B-D,** *D-min* was averaged in 20-trial bins. The insets in each plot show the averaged data for all six practice days.

Performance was characterized by the average distance error *D-min*. Fig 3A shows one subject’s actual *D-min* (black hollow circles) and manipulated (red solid circles) *D-min* across six days of practice (DEA-2.0 group). As was typical, this subject decreased *D-min* across the first two days at a rate consistent with previous experiments on the same task [2, 22, 38, 39]. At the end of the third day the subject’s performance plateaued. This leveling-off in error reduction was verified for each individual subject by linear regression on the binned *D-min* values on Day 3 (the first 12-trial bin was excluded to remove initial transients). None of the regression slopes differed from zero for all subjects in all groups (*p* > .10 for all groups, except *p* = .07 for DEA 2.0).

To test *Hypothesis 1*—amplifying perceived errors improves task performance—changes in *D-min* were examined over the last three days of practice. For groups receiving error amplification (either DEA or SEA), *D-min* decreased at a faster rate than in the control group (Fig 3). This effect was more pronounced at higher amplification levels. Specifically, the linear regression slopes over Day 4 to Day 6 for DEA-2.0, SEA-2.0, DEA-2.5, and SEA-2.5 were more negative than slopes for the group of control subjects (*p* < .003 for all comparisons). The slopes for groups DEA-1.5 and SEA-1.5 were also negative, but the difference compared to the control group did not reach statistical significance (*p* = .08 and .09, respectively).

**Fig 4:**
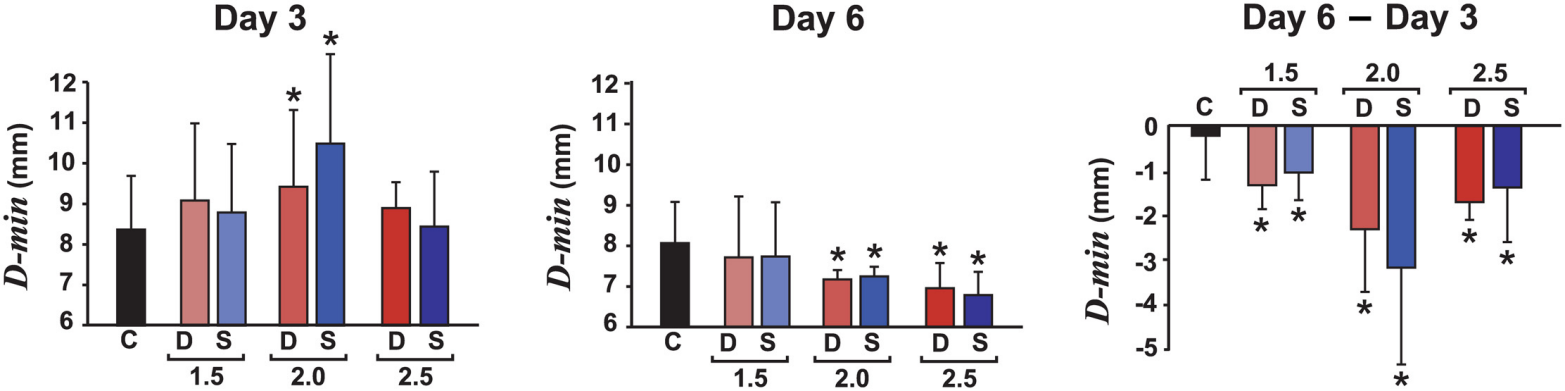
Summary data showing that error amplification decreased the error *D-min*. Bars show: Day 3 (before error amplification), Day 6 (last day of practice with error amplification), and the change from Day 6 –Day 3. Participants received either deterministic (D; red) or stochastic (S; blue) error amplification on the last three practice days with amplification factors of 1.5, 2.0, or 2.5. The control group (C; black) received no error amplification. Error bars: between-subjects standard deviation. *Groups significantly different than the control at *p* < .05.

To further test *Hypothesis 1*, *D-min* was compared across groups, separately on Day 3 and Day 6, i.e., before and after error amplification, and its change from Day 3 to Day 6. On Day 3 (Fig 4, left) *D-min* in four of the experimental groups did not differ from controls; DEA-2.0 and SEA-2.0 had larger errors probably due to random group differences (*p* = .019 and *p* < .001, respectively). On Day 6, all groups with amplification higher than 1.5 had a significantly smaller error than controls (*p* < .01 for all). For the change across practice, all experimental groups showed greater decreases in *D-min* compared to the control group (*p* < .001 for *A ≥* 2.0; *p* = .021 for DEA-1.5 and *p* = .043 for SEA-1.5). The groups with 2.0 amplification showed particularly large changes due to their initially higher errors. Overall, these results demonstrate a benefit conferred by error amplification, particularly for larger amplification gains.

To test *Hypothesis 2*—stochastic amplification of perceived errors improves task performance more than deterministic error amplification—the slopes of the change in *D-min* over the last three days were compared between DEA and SEA at each amplification level. There were no differences in the slopes between the DEA and SEA groups at any error amplification level (*p* > .05). When the groups were compared before and after error amplification, i.e. separately on Day 3 and Day 6, there were no significant differences in *D-min* between deterministic and stochastic amplifications (DEA vs. SEA) at any of the amplification magnitudes (*p* > .55 for all comparisons).

#### Release velocity

**Fig 5:**
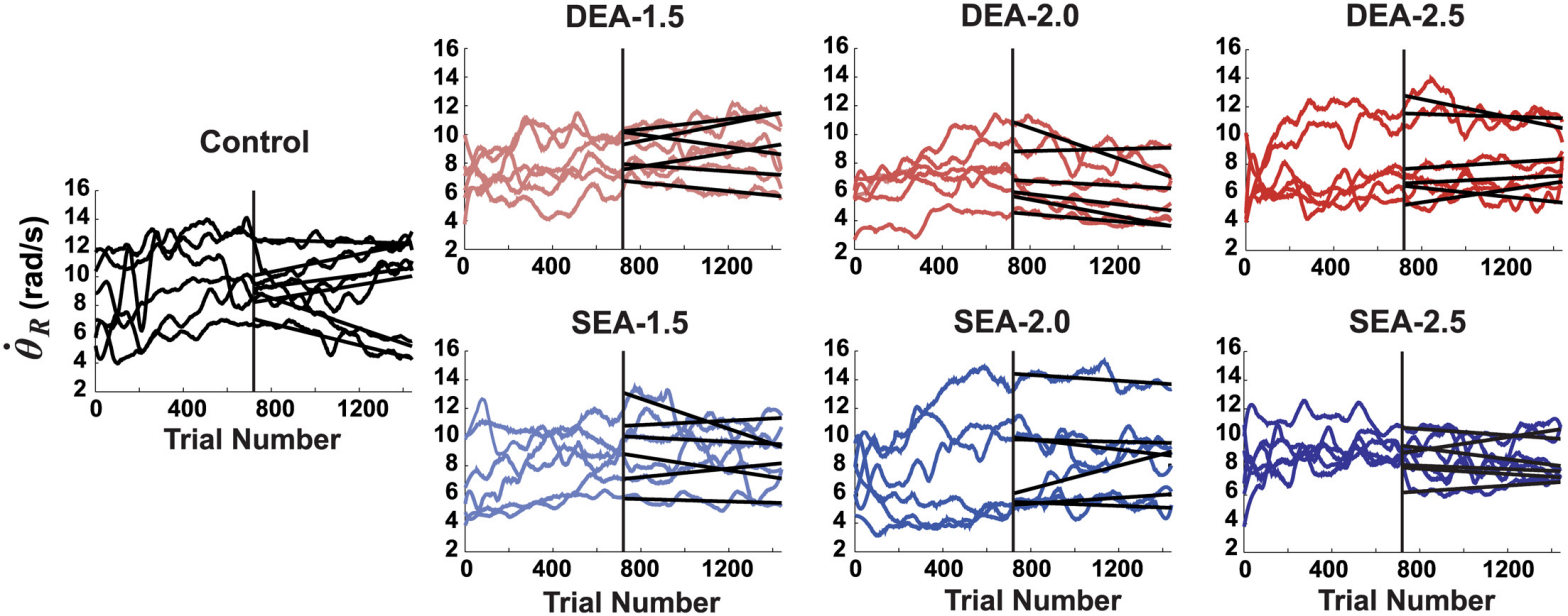
Absence of systematic changes in the release velocity with practice. The release velocities for each subject in each group are shown for all practice trials. Linear fits to the last three days are also shown. There was no systematic decreases in velocity as would be expected from speed-accuracy trade-off. Data were smoothed with a third-order Savitzky-Golay filter with frame size = 101.

To assess whether the systematic decrease in error was brought about by other strategic changes in movement velocity, release velocity was evaluated. In particular, subjects may have decreased velocity to thereby decrease variability, i.e. traded speed for accuracy. The target constellation with the specific shape of the solution manifold made this trade-off possible. The data presented in Fig 5 shows the release velocities of all groups across the six days of practice. The figure highlights the trends with regression lines over the last three days of practice. In all but one case, the slopes showed little systematic variation. Only the slopes for the DEA-2.0 group were significantly more negative than the control group (*p* = .016), and this was due to a strong trend observed in one subject. Hence, there was no support for the speed-accuracy trade-off as a mechanism to lower the observed variability.

#### Release velocity variability

**Fig 6:**
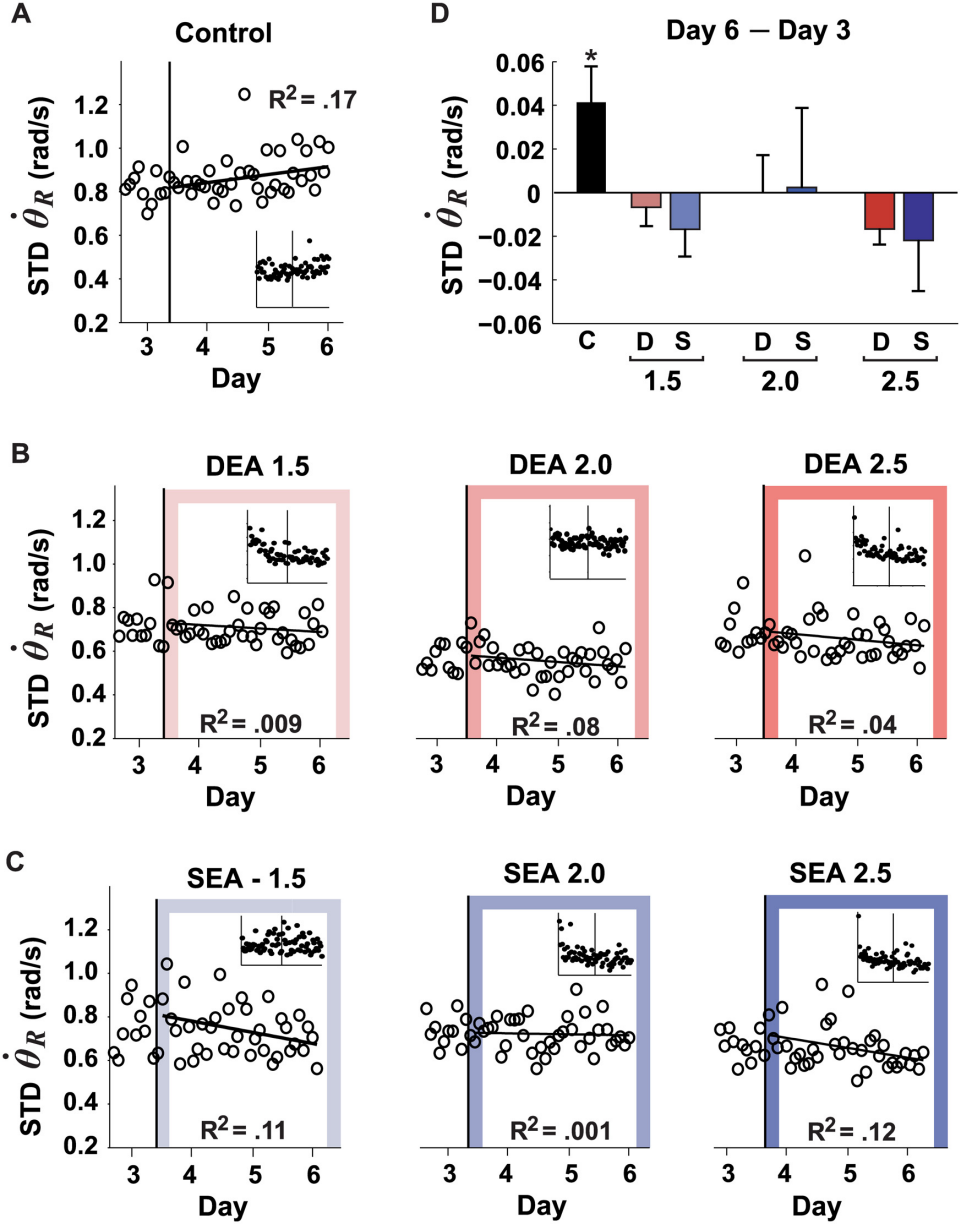
Changes in release velocity variability with practice. The six error amplification groups showed no significant changes. The control group slightly increased the variability of their release velocity with continued practice. Panels **A-C** shows the standard deviation of the release velocity for the last day of practice with no manipulation (Day 3) and three subsequent days (Day 4–6) in which subjects received either no error amplification (controls; panel **A**) or different magnitudes of deterministic or stochastic error amplification (DEA or SEA, respectively; panels **B** and **C**). Data were averaged in 20-trial bins. The insets in each plot show the averaged data for all six practice days. Panel **D** shows the change from Day 6 –Day 3. Error bars: between-subjects standard deviation. *Indicated that the change is significantly different from zero *p* < .05. C = control (no manipulation; black); D = deterministic error amplification (red), S = stochastic error amplification (blue).

Another computational avenue to decrease variability in task performance is to channel irreducible noise into task-irrelevant dimensions. In this task constellation, subjects could have increased variability in release velocity without experiencing any negative effect on overall task error. As shown in Fig 6, the control group indeed increased the variability of their release velocity with continued practice, after release angle variability had reached a plateau (the change between Day 3 and 6 was significantly different from zero; Fig 6D, *p* = .004). In contrast, none of the error amplification groups showed any significant change (not different from zero; *p* > .087 for all). When compared with the error amplification groups, the control group had a larger increase in release velocity variability than all error amplification groups (*p* < .012 for all comparisons). This result gave clear evidence that redirecting undesired noise into redundant task dimensions was not pursued by the error amplification groups. Rather, variability decreased when practicing with error amplification.

### Effects of Error Amplification on Correction Gain and Motor Noise (Hypotheses 3–5)

To further tease apart potential mechanisms underlying the decrease in overt variability, the time series of release variables were examined via stochastic learning models. In particular, three models were used to extricate the contribution of the error correction gain from random noise sources. Model parameters were estimated before and after error amplification using system identification. Parameter estimation was conducted separately for each block of 60 trials; there was a total of 1008 blocks across all days and subjects (42 subjects, 6 days, 4 blocks on each day). The 60 trials presented a sufficiently long time series that also avoided potential drifts that may have otherwise confounded the parameter estimation.

**Fig 7:**
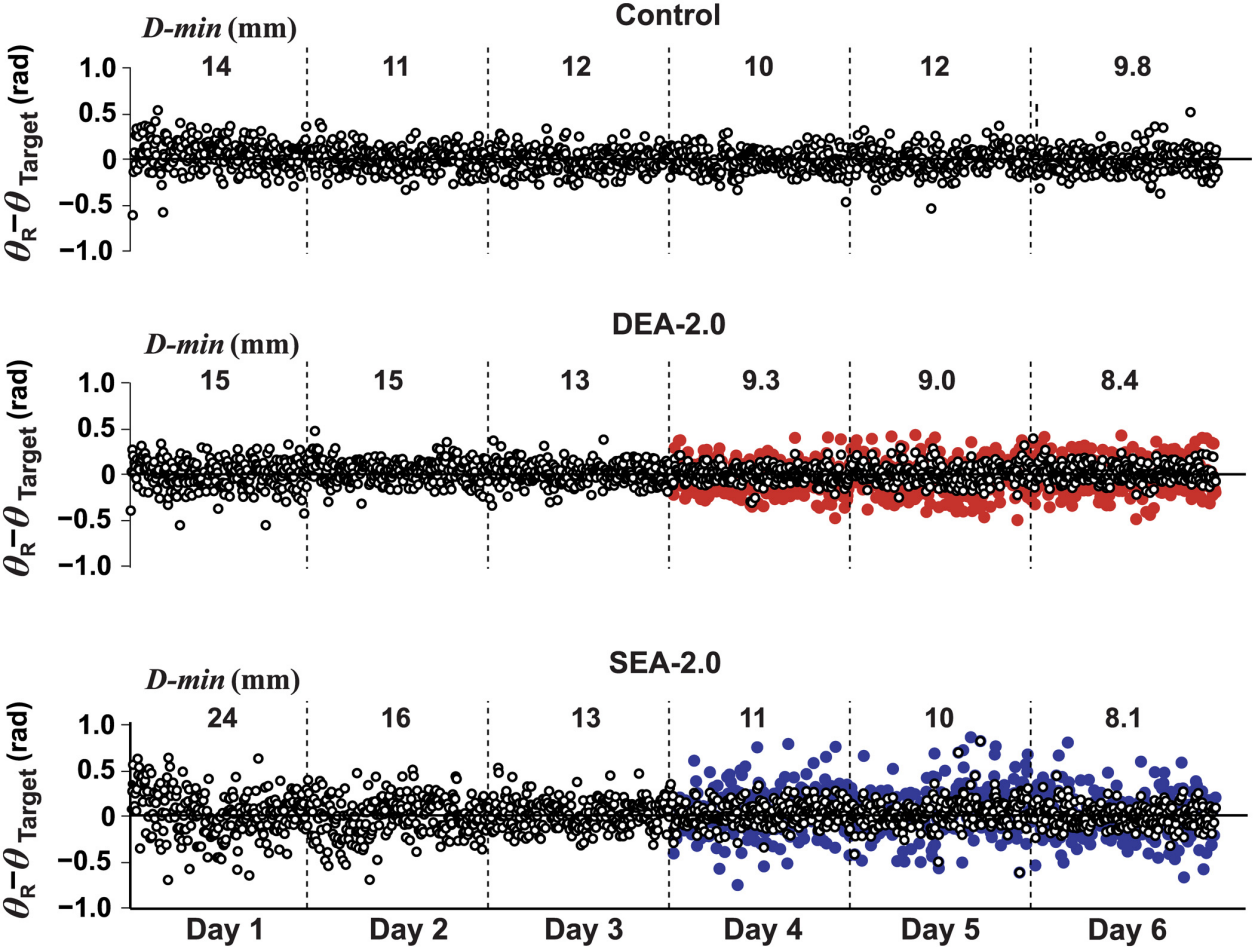
Error in release angle across six days of practice for three exemplar subjects. Shown are data for one subject from the control group (top) and deterministic (middle) and stochastic (bottom) error amplification groups (DEA and SEA, respectively). The amplified errors on the last three days are shown by the closed colored circles (DEA: red; SEA: blue). The numbers above each day show the average distance error *D-min* for each of the six days.

Examples of the raw data of angular error *e* = *θ*_*R*_ − *θ*_*Target*_ used in the system identification are displayed for three subjects in different groups in Fig 7. Note this error could be both positive and negative, unlike the distance error *D-min*. In addition, the deviation of the release angle to the optimal angle could be reduced to zero. The open black circles denote errors in unmanipulated trials; the closed colored circles represent the amplified errors as subjects saw on the screen. The SEA condition had clearly a wider range of amplified errors than DEA. The long sequence of 1440 trials showed a very gradual, almost invisible change in release angle error; nevertheless, the average values showed a clear reduction across days as previously seen in *D-min* (Fig 3).

#### Error correction gain

**Fig 8:**
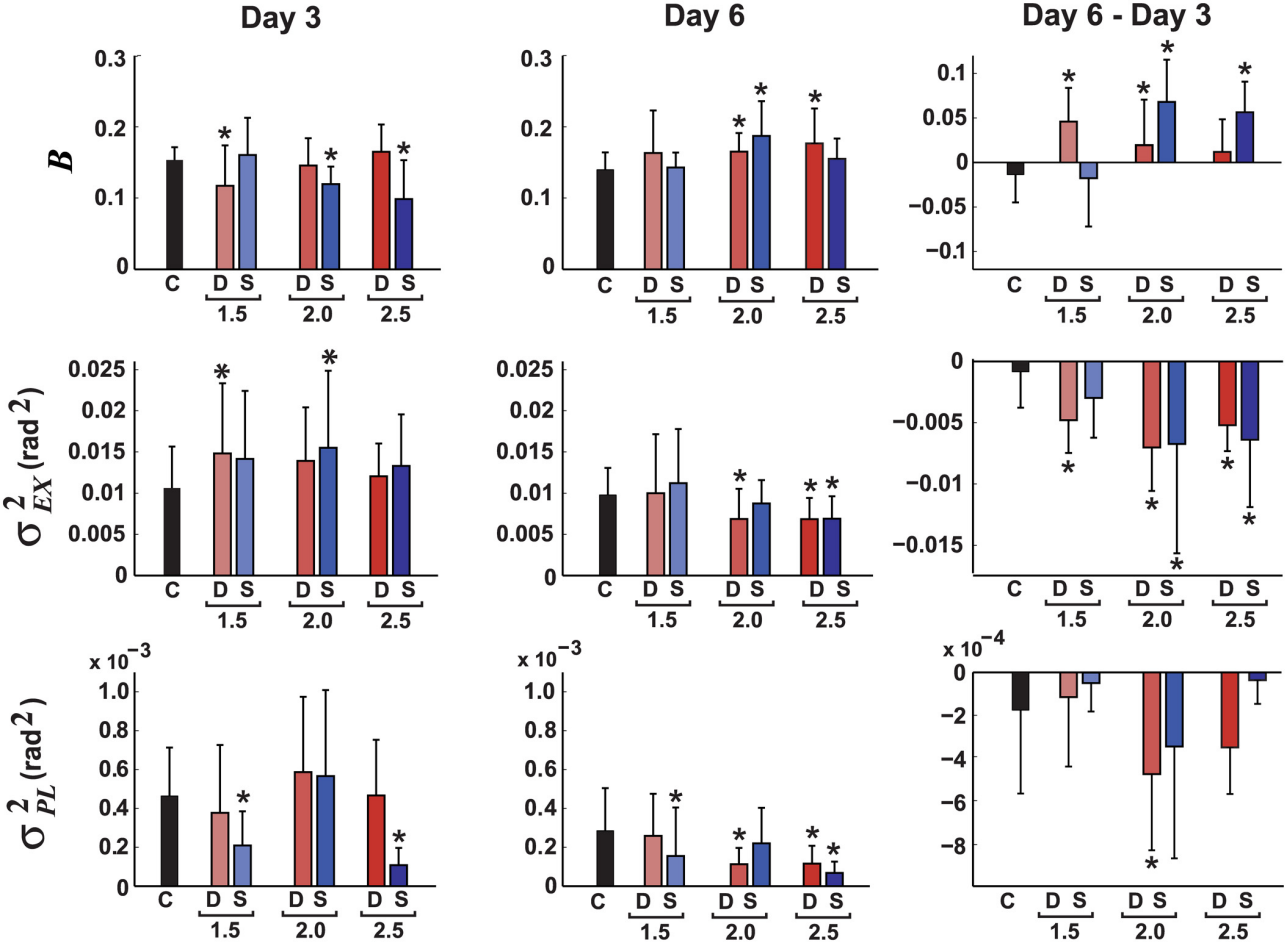
Error amplification led to modest increases in participants’ error correction gain *B*, but larger decreases in execution and planning noise variance, *σ*_*EX*_^2^ and *σ*_*PL*_^2^, respectively. Bars show: Day 3 (before error amplification), Day 6 (last day of practice with error amplification), and the change from Day 6 –Day 3. Error bars show the between-subjects standard deviation. The manipulation conditions were: C = control (black); D = deterministic error amplification (red), S = stochastic error amplification (blue). *Groups significantly different than the control at *p* < 0.05.

The effects of error amplification on the participants’ correction gains *B* were examined to test *Hypothesis 3* –that error amplification increases the size of error corrections. The results are shown in Fig 8. On Day 3, DEA-1.5, SEA-2.0, and SEA-2.5 had smaller *B* than controls (*p* < = .01 for all). On Day 6, DEA-2.0, DEA-2.5, and SEA-2.0 had a larger *B* than controls (*p* < = .05 for all). Comparing the change in *B* from Day 3 to Day 6, most error amplification groups showed an increase in *B* compared to controls (*p* < .05), except the SEA-1.5 (*p* = .80) and DEA-2.5 (*p* = .12) groups that did not differ from controls. Overall, these results provided moderate support for *Hypothesis 3*, i.e. error amplification tended to increase *B* at the higher amplification levels.

To test for differences among amplification levels, the three DEA groups were compared with each other, and the three SEA groups were compared with each other. The results were modest: For the DEA comparison, there were no group differences on Day 6. The change from Day 3 to Day 6 showed that DEA-1.5 increased *B* more than SEA-2.5 (*p* = .03). For the SEA comparisons, on Day 6 *B* for SEA-2.0 was greater than for SEA-1.5 (*p* < .001) and greater than SEA-2.5 (*p* < .001). For the change in *B*, SEA-2.0 and SEA-2.5 increased *B* more than SEA-1.5 (*p* < .001 for both comparisons). In general, there was some evidence for an increase in the correction gain *B* for higher amplification values with SEA, but the opposite was the case for DEA. More importantly, the actual values were very small.

#### Neuromotor noise

To test *Hypothesis 4* –error amplification reduces intrinsic neuromotor noise—the level of *σ*_*EX*_^2^ and *σ*_*PL*_^2^ for each group was compared with the control group. The results for *σ*_*EX*_^2^ are shown in Fig 8 with the following detailed statistical comparisons. On Day 3, DEA-1.5 and SEA-2.0 had larger *σ*_*EX*_^2^ than controls (*p* = .05 and .02, respectively). On Day 6, DEA-2.0, DEA-2.5, and SEA-2.5 had smaller *σ*_*EX*_^2^ than controls (*p* = .04 for all). For the change from Day 3–6, all groups except SEA-1.5 showed more change in *σ*_*EX*_^2^ than controls (*p* = .004 for DEA-1.5; *p* < .001 for others). The results for *σ*_*PL*_^2^ gave a similar picture. On Day 3, SEA-1.5 and 2.5 had smaller *σ*_*PL*_^2^ than controls (*p* = .01 and *p* < .001, respectively). On Day 6, DEA-2.0, DEA-2.5, SEA-1.5, and SEA-2.5 had smaller *σ*_*PL*_^2^ than controls (*p* = < .001 for all except SEA-1.5 with *p* = .02). For the change from Day 3–6, DEA-2.0 had a greater reduction in *σ*_*PL*_^2^ than controls (*p* = .004). Collectively, these results show that there was a reduction in neuromotor noise both in *σ*_*EX*_^2^ and to a lesser degree in *σ*_*PL*_^2^, supporting *Hypothesis 4*.

To test *Hypothesis 5*—stochastic amplification reduces noise more than deterministic error amplification—*σ*_*EX*_^2^ and *σ*_*PL*_^2^ was compared for each pair of DEA and SEA groups within an error amplification level. For *σ*_*EX*_^2^, there were no significant DEA vs. SEA differences at any amplification level for Day 3, Day 6, and the change from Day 3–6. However, for *σ*_*PL*_, on Day 6 DEA-2.5 was more noisy than SEA-2.5 (*p* = .003). There were no DEA vs. SEA differences in *σ*_*PL*_^2^ on Day 6, but for the change from Day 3–6, DEA-2.5 decreased noise more than DEA-2.5 (*p* = .002). Overall, these results did not support *Hypothesis 5*, as there were no systematic differences between the effects of DEA vs. SEA on neuromotor noise.

To test for possible differences in *σ*_*EX*_^2^ and *σ*_*PL*_^2^ among error amplification levels, each DEA group was compared with each other, and each SEA group was compared with each other. The results for *σ*_*EX*_^2^ are presented first. For DEA there were no Day 3 differences, but on Day 6 DEA-1.5 had greater noise than DEA-2.0 (*p* = .03) and DEA-2.5 (*p* = .02). The change across Day 3–6 rendered no significant results. For SEA, there were no Day 3 differences, only on Day 6 SEA-1.5 had greater noise than SEA-1.5 (*p* = .002). For the change SEA-1.5 decreased noise less than SEA 2.0 (*p* = .010) and SEA-2.5 (*p* = .020). Now the results for *σ*_*PL*_^2^ are presented. For DEA on Day 3, DEA-1.5 had smaller *σ*_*PL*_^2^ than DEA-2.0: (*p* = .02). On Day 6 DEA-2.0 and DEA-2.5 had smaller *σ*_*PL*_^2^ than DEA-2.0 (*p* = .007, and *p* = .01, respectively). For the change from Day 3–6, *σ*_*PL*_^2^ for DEA-2.0 and DEA-2.5 were smaller than DEA-1.5 (*p* < .001 and *p* = .03, respectively). For SEA on Day 3, SEA-1.5 and DEA-2.5 had smaller *σ*_*PL*_^2^ than SEA-2.0 (*p* < .001 for both comparisons). For Day 6, SEA-2.5 had a smaller *σ*_*PL*_^2^ than SEA-2.0. For the change from Day 3-Day 6, SEA-2.0 had a greater reduction in *σ*_*PL*_^2^ compared to SEA-2.0 (*p* = .005) and SEA-2.5 (*p* = .003). Overall, these results show that the reduction in neuromotor noise associated with error amplification was dependent on the magnitude of amplification.

#### Noise trends

**Fig 9:**
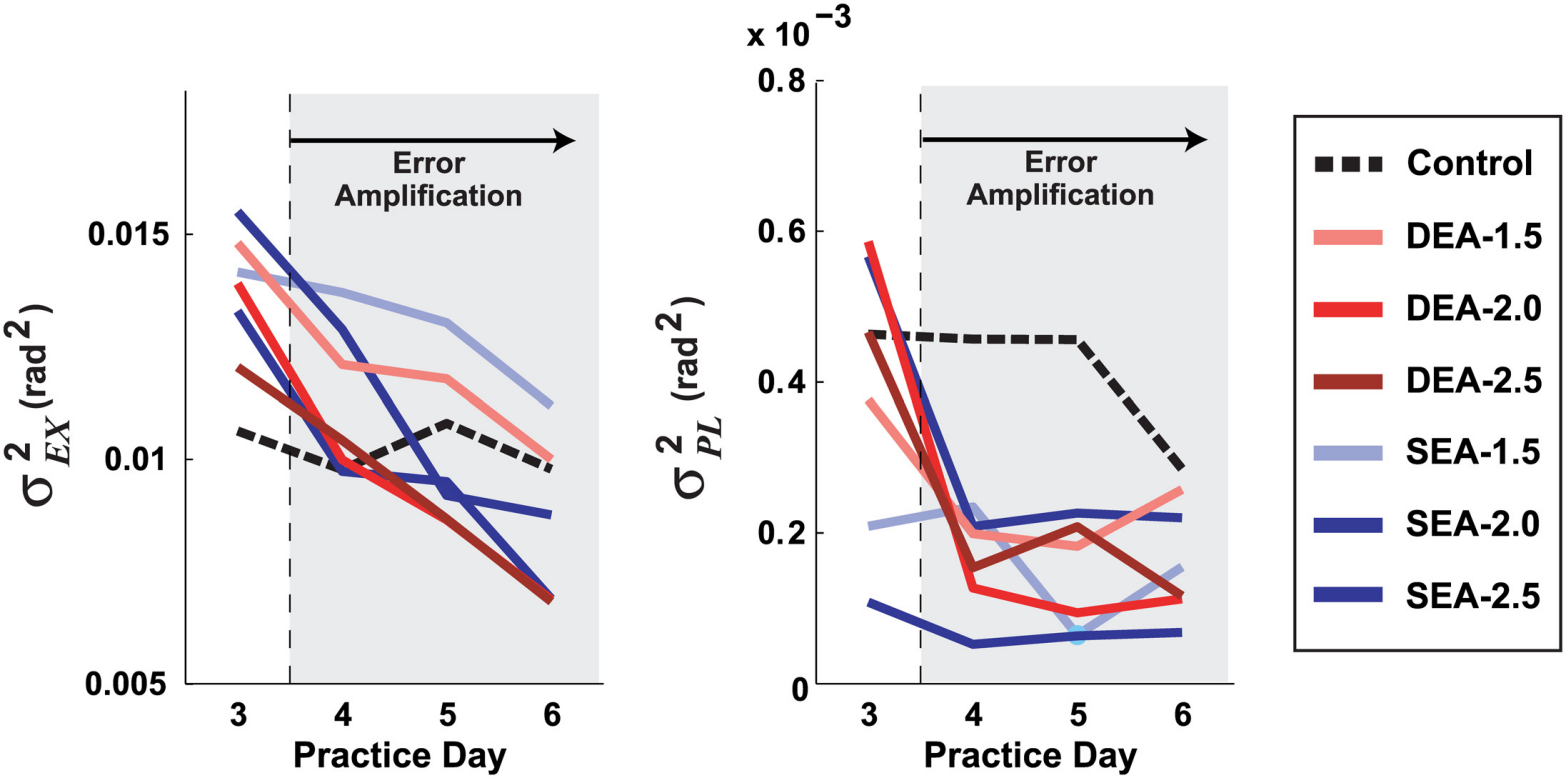
With error amplification execution noise *σ*_*EX*_^2^ decreased consistently; planning noise *σ*_*PL*_^2^ dropped more abruptly. The estimated *σ*_*EX*_^2^ and *σ*_*PL*_^2^ are shown for the last day without manipulation (Day 3), and the three days with error amplification (Day 4–6). The data are averaged across all subjects in each group. Three levels of amplification were used for each DEA and SEA group: 1.5, 2.0, and 2.5.

Of the estimated parameters, the change in noise over practice showed the clearest trends. Plotting the change in *σ*_*EX*_^2^ and *σ*_*PL*_^2^ for the last day before amplification, and each of the three days with the amplification, presents a compelling picture of the effects of error amplification on neuromotor noise. Fig 9 shows a steady decline in *σ*_*EX*_^2^ for all error amplification groups, but not for the control group. For *σ*_*PL*_^2^, there was an abrupt drop immediately after the manipulation was applied, but then little change for the next two days.

#### System identification results for Models 1 and 2

The results of the first two models are summarized in S2 Appendix. In brief, there were trends showing an increase in *B* values with practice in the amplified conditions, but they were no consistent results across conditions. The *B* values for Model 1 were close to zero. For Model 2 there were many blocks that rendered negative *K* values that could not be interpreted. Despite these irregularities in the estimates for *B* and *K*, both models showed a decrease in noise with error amplification, particularly at the higher amplification levels, consistent with Model 3.

## Discussion

This study demonstrated how error amplification affected error and variability in the performance of a perceptual-motor task. Performance at asymptote was examined to downplay the error correction processes that naturally predominate in the early stages of learning. Results showed that visual amplification of error achieved decreases in mean error, even after subjects had reached a performance plateau. A model-based analysis of trial-by-trial error corrections revealed that error amplification improved performance mainly by reducing the level of neuromotor noise, rather than by modifying the error correction process itself. Although the observed decrease in the random components of motor behavior cannot be attributed to any specific physiological structure at present, this study represents a critical step towards understanding how error amplification can benefit motor performance. The finding that improvements can be attained after subjects had reached an asymptote is significant for therapy, when patients are unable to make any further progress or may be stuck in a “local minimum”.

### Effects of Error Amplification on Task Performance

All participants underwent extensive practice over three days (720 trials) until their performance had reached a plateau. Six experimental groups that continued to practice for three more days with visually amplified errors (another 720 trials) further decreased their errors, while the control group did not. This improvement was seen for both deterministic and stochastic amplification. Corroborating previous demonstrations [13–19], these findings present clear evidence that amplifying perceived errors improved task performance (*Hypothesis 1*).

Extending prior work, three different amplification gains were compared to parametrically assess the effect of error augmentation magnitude. As expected, the lowest gain of 1.5 was not effective, neither in stochastic nor in deterministic form. The two larger gains of 2.0 and 2.5 elicited improvements by virtue of a decreased mean absolute error. Although in some cases the gain of 2.0 elicited the greatest improvements, this could have been because this group was worse than others prior to the manipulation. The highest amplification gain 2.5 did not produce any “instability”, as speculated previously. Wei et al. [18, 19] stated that for their perturbed reaching task an error amplification gain of 3.1 would lead to overcorrection and instability based on an error updating model [20], a result that was supported by their experimental data. While the higher amplifications in the present task were successful, one must keep in mind that the specific numerical values are most likely task-dependent.

We also posited that the addition of stochastic noise may enhance the effect of amplification (*Hypothesis 2*). However, the expectation that the additional noise would add “pressure” to reduce the variability of their performance was not confirmed. We speculate that subjects may simply have “averaged” over the noisy errors, and were therefore insensitive to the immediate error information presented after each trial.

### Effects of Error Amplification on Error Correction Gain

For any account of learning, there are two primary options to improve overt errors in the performance variable: optimize the error correction gain and/or reduce the internal noise variance. To tease these two options apart from the overt variability of the task performance, the present study applied system identification procedures based on three stochastic iterative learning models. All three models included two stages, motivated by previous electrophysiological research that identified significant contributions of noise in the planning processes preceding movement, distinct from execution [40, 41]. A model with two stages of noise was also supported by previous behavioral and modeling studies that assessed structure in the trial-by-trial changes in an aiming task and the skittles task [21, 22].

The system identification results showed that error amplification elicited a small increase in the correction gain or learning rate *B*, which supports *Hypothesis 3*, and this increase became larger with larger amplification magnitudes (for SEA, not DEA). This result was provided by Model 3, which was deemed the most appropriate of the three models tested. Although subjects increased the size of their corrections, the increases remained modest, averaging about 5% at most, and fell significantly short of the amplification factors. If participants had fully adjusted the size of their error corrections to the amplification, then there should have been increases of 50%, 100%, and 150% for the three error amplification levels (1.5, 2.0, 2.5, respectively). This under-compensation agrees with prior work showing that humans typically do not respond proportionately to errors, i.e. they respond to larger errors less than would be expected [42]. The relatively small adjustments in participants’ error correction gains in response to error amplification suggest that this gain adjustment was not the prime driver of the observed improvements in performance.

The values for *B* were mostly between 0.1 and 0.2 (10%–20% of error corrected), which is somewhat lower than other studies, which have reported correction gains of about 0.38 in an aiming task [21] and in the range of 0.20–0.50 for a reaching task [36]. The lower values in the present study could arise from the fact that the actions were well-practiced. However, note that *B* results depend significantly on the choice of the model; therefore, it is important to compare different models to assess their suitability [e.g. 43]. In our study, Model 1 with only a single noise source and Model 2 with two noise sources were not in agreement (see S2 Appendix). By the last day of practice, both models estimated that half of the error amplification groups had higher *B* values than the control group, but these groups were not the same. For Model 2 the *B* values were generally large compared to Model 1 where the gains were often less than 0.1. Further, when noise is part of the dynamics, parameter estimation methods may produce a bias [37]. Given these discrepancies and potential estimation problems, we refrained from further interpreting the gains. Note that amplifying the errors in the modeling itself would not alter the relative changes in *B*, but would only increase the absolute values of *B* by a factor equal to the amplification gain (assuming subjects fully respond to the amplified errors).

### Effects of Error Amplification on Neuromotor Noise

The most robust result of the system identification, consistent in all three models, was that error amplification reduced the noise sources, supporting *Hypothesis 4* (see also S2 Appendix). While execution noise showed a steady decline after error amplification was applied, planning noise declined more abruptly and reached a plateau (Fig 9). Although planning noise remained steady in the control group for most of their practice, there was a precipitous drop on the very last practice day. This behavior was consistent across all subjects in this group. Thus, even without error amplification, planning noise may eventually decline, only at a later stage. This observation may also signal that performance improvements due to error amplification may just accelerate internal learning processes, and not introduce new mechanisms. Related work has shown that even without any external rewards, individuals can eventually reach the same performance level as those that are rewarded, but do so at a slower time scale [25, 44].

While the variance of planning noise was much smaller than execution noise, this does not necessarily imply that planning noise had a negligible impact. For example, for the control group on Day 3, the two noise variances averaged to about 0.00045 and 0.011 rad^2^. While seemingly small, this equated to standard deviations of about 1.2° and 6.0° for planning and execution noise, respectively. Thus, the planning noise contributed about 21% to the variability in the release angle. Although it was also hypothesized that that stochastic amplification would reduce noise more than deterministic error amplification (*Hypothesis 5*), this hypothesis was not supported. Potentially, participants did not make trial-by-trial adjustments to their actions in response to the visually amplified errors, but instead may average across multiple trials. It should also be kept in mind that stochastic amplification only added one random number to the release angle of each throw; their arm movements leading up to the release were not continuously perturbed. Hence, the added randomness may have been lost on the subjects.

### Control Mechanisms Leading to Lower Neuromotor Noise

A decrease in variability is often achieved by slowing down movement, i.e. a decrease in speed is traded for an increase in accuracy [45, 46]. The speed-accuracy trade-off has been proposed as a general signature of learning; a more skilled individual can execute movements faster and with greater precision [46]. Further, it is generally believed that signal-dependent noise is a basic property of neuronal signaling [47]. The effects of this noise are readily seen during isometric force production, with the variability of the exerted force increasing as a multiple of the force [48]. For dynamic tasks and trajectories this manifests itself as velocity-dependent noise. Counter to these expectations, participants in the present study did not exhibit systematic decreases in their release velocity across practice, consistent with previous results on a similar task [24]. This suggests that other avenues were available for the neuromotor system to reduce its overt fluctuations.

Previous research on the same throwing task highlighted three conceptually different avenues to decrease observed variability. Subjects can: 1) find solutions that are error-tolerant, 2) co-vary execution variables to minimize the effect of variability on the task result, and 3) reduce the variance of noise [2, 23, 39, 49, 50]. This work showed that error tolerance was achieved very early in practice, while co-variation was a computational strategy that was gradually exploited with practice. Noise remained the component least accessible to even extended practice [2]. In the present study, which included a relatively long practice schedule, tolerance was of little relevance after the first day of practice. Co-variation between the release angle and velocity could have been exploited to channel noise into task-irrelevant dimension, without necessarily reducing the overall amplitude of noise sources. In the present task, release velocity presented such a task-irrelevant dimension. However, counter to expectation, variability in release velocity did not increase concomitant to the decrease in angle variability. Hence, this computational strategy could not explain the lowering of noise. To improve performance subjects could only reduce the noise component.

### Limited Redundancy

The present target constellation rendered a very specific solution manifold that gives release velocity little contribution to the observed error. However, velocity is not entirely irrelevant. Both release angle and velocity are needed to calculate the ball trajectory. Importantly, the error sensitivity of throws with different velocities changes, as seen by a slight broadening of the neighborhood with low errors at higher velocities (Fig 1C). Subjects can visually distinguish between different release velocities as they lead to ball trajectories with different elliptic paths (Fig 1B). As shown in a previous study, subjects seek those more error-tolerant velocities and do not seek the lowest velocity that might be expected due to the least amount of signal-dependent noise [24]. Finally, small changes in target locations will make release velocity an important determinant of the distance error. Hence, the velocity dimension is not quite as redundant as it appears at first sight.

### Physiological Mechanisms to Decrease Neuromotor Noise

How might subjects have reduced overt noise and possibly physiological noise in response to error amplification? This question is partly motivated by a known mechanisms in songbird learning, where the nervous system can purposefully inject noise into the learning process during early-learning, and then reduce these self-induced perturbations after learning [51]. Might the human nervous system use noise in a similar way? Humans can mechanically reduce the influence of noise on task performance with antagonistic co-activation [52–54]. However, while antagonistic co-activation may increase in early learning, it typically reduces with practice [55]. In the present study, signal-dependent noise has been ruled out as an explanation because the release velocity did not systematically decrease with practice (assuming that velocity presents the “signal”) [47]. Further, there is some evidence that this noise can change with alterations in the physiological properties of motor units, such as reported in aging muscles [56], but it remains unknown whether this noise can be altered on a shorter time scale. One final speculation is derived from studies on the effect of neuromodulators, such as serotonin or norepinephrine, on motor neuron excitability [5, 6, 57]. Animal studies provided significant evidence that the descending drive to muscle contractions is gain-controlled to modulate the required output force. One study on humans showed that force variability increased after the brainstem–spinal cord neuromodulatory system was up-regulated [6]. A complex interplay of neuromodulators can excite or inhibit spinal cord excitability and thereby match precision demands of motor behavior. It is possible that this gain control is modifiable via error amplification. Evidently, more research is needed to solidify these conjectures.

### Other Mechanisms Contributing to Improved Performance

As noted earlier, there could be other factors, such as enhanced perception and correction of errors and motivational factors that may contribute to improved performance with error amplification [18, 19]. In terms of perception, it could be that increasingly smaller errors become simply too small to detect and amplifying error resolves this problem. However, the present results do not support this conjecture: if the control group had been unable to detect their errors, then their error correction gains should have been close to zero. Counter to this expectation, although small, their gains remained significantly above zero, even after six days of practice. This only remains a possibility if one conjectures that subjects corrected their predicted errors based on internal models of the task.

### Considerations and Limitations of the Analysis of Temporal Structure

Temporal structure of data can be analyzed with numerous methods, ranging from simple autocorrelation and other linear autoregressive methods to nonlinear methods, such as entropy analysis [58] or recurrence quantification [59]. Autoregressive methods maintain temporal connections and are strictly linear analyses. On the other hand, more sophisticated nonlinear methods, such as multi-scale entropy analyses or recurrence quantification, are useful for more continuous time series, as seen in postural control or heart rate [60–62]. We opted to use iterative learning models to analyze trial-to-trial sequences, where error correction processes explicitly link successive trials and there are explicit parameters of the two processes in focus. However, stochastic iterative models are clearly also not free of limitations.

First and common to all analysis is that temporal analyses select one single variable from a complex movement system. While the neuromotor system is unquestionably high-dimensional and has a highly distributed neural network, task success in the current task was described by a single kinematic variable, angle at ball release. This variable lent itself to be mapped on the single model variable. Second, in the task subjects viewed the distance error between their ball trajectory and the target, whereas in the model the error information was the difference in release angle to the optimal angle. Given the parabolic relation between the two, this may have influenced the result, lowering the estimates of *B*. However, after two days of practice most subjects were close to the optimal release angle, and therefore operated in a regime where the relation was close to linear. Probably more critical is that the mapping of the perceived error in workspace to the error in release angle may include further inaccuracies.

Another note of caution relates to the models themselves. The comparative analyses of three basic learning models highlighted that parameter estimates are very sensitive to the structure of the model. For example, all three models assumed additive noise. It is conceivable that the learning mechanisms include multiplicative noise. As we do not know the “true” model, results of these estimations should be interpreted with great care. Further, parameter estimation of a stochastic model has computational pitfalls, and the seemingly straightforward estimates of error correction gains become biased when noise is included [37], as we showed in S1 Appendix. We therefore only interpreted the changes in noise that was in agreement in all three models. We also tried to rule out alternative explanations and apply caution in the interpretation. Evidently, stochastic learning models with different forms of noise will have to be a topic for further research in motor neuroscience.

Finally, there is always the question of generalization: do the observed effects of error amplification extend to other motor tasks? For example, do the findings also hold for continuous tasks that involve tracking? How could error amplification be applied without a virtual interface? Do the results from the virtually controlled task generalize to real-world skittles throwing in 3D? These critical questions are clearly not confined to our study, but to almost all controlled experimental studies. While it is difficult to draw any definitive conclusions within the scope of a single study, the present results add to existing evidence. Prior studies have shown error amplification benefits in different reaching tasks [15–19] and a pinball-type task [13]. Further, beneficial effects of error amplification have been reported for different sensory modalities, including visual [16–18] and haptic feedback [10, 12]. Although these studies did not separate improvements due to noise reduction from changes in error correction, it is plausible that similar effects may be found. Of course, further research should substantiate this possibility.

### Conclusions

The main results of this study was that error amplification elicited continued improvements in performance that otherwise plateaued, and this effect was mainly driven by a reduction in neuromotor noise. As such, error amplification presents a way of stimulating continued improvements in motor performance. The results challenge the assumption that neuromotor noise is invariant and inaccessible to behavioral interventions. Error amplification has the potential to become an effective intervention for improving motor performance in physical therapy and neuro-rehabilitation to improve motor function. This can be of special benefit for those who have reached a performance plateau, ranging from elite athletes, to patients with neuromuscular disorders, and to the elderly.

## Supporting Information

S1 Appendix Results of model validation and details of approach for reducing estimation bias. (PDF)

S2 Appendix Results of system identification for all models. (PDF)

S1 Dataset Supporting Data. (ZIP)
