# Supplementary material for: Neuromotor Noise Is Malleable by Amplifying Perceived Errors
Source: PLoS Comput Biol. 2016 Aug 4;12(8):e1005044. doi: 10.1371/journal.pcbi.1005044 (PMC4973920; doi:10.1371/journal.pcbi.1005044)
Supplement: S1 Appendix — (PDF) [file pcbi.1005044.s001.pdf]

Appendix 1

Results of Model Validation and Details of Approach for Reducing Estimation Bias

Validation of System Identification for Models 1 and 2

Monte-Carlo simulations were performed to validate the accuracy and precision of the system identification of the learning Models 1 and 2. Model 1 (Equations 12-14; main text) parameters included the error correction gain B and the noise variance σ^2. In Model 2 (Equations 17-19; main text), the additional parameter K specified the ratio between the magnitude of planning and execution noise (Equation 19; main text). Note that Model 1 is a special case of Model 2 with $K = 1$.

Simulations were run for different parameter combinations of B, K, and σ, for a length of 60 iterations each, corresponding to the 60 trials per block in the experiment. One-hundred simulations were run for each parameter combination. The MATLAB System Identification Toolbox was then used to estimate B, K, and σ^2 for the simulated data, in the same way as the identification of the experimental data described in the main text. The results of the validation procedure are shown in Figure A1.1.

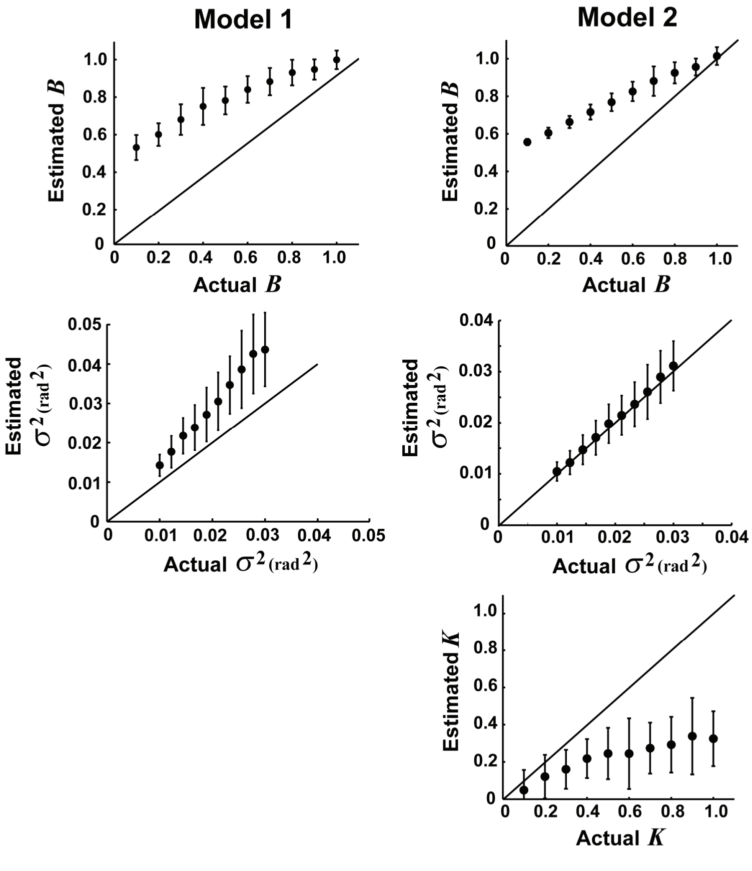

Figure A1.1. *Validation results for Models 1 and 2. The line shows the true values used for the simulations, the data points show the estimated values, and the error bars represent the standard deviation of 100 simulation runs. For Model 1 the B results are obtained with fixed noise magnitude: σ^2=0.02; the results of the noise were run with B=0.2. For Model 2 the B results were obtained with K=0.4 and σ^2=0.02; the K results were obtained with fixed B=0.2 and σ^2=0.02.*

The validation results show that B was overestimated across the entire range in both Model 1 and 2. For Model 1, σ^2 had a positive bias, but there was no bias in σ^2 for Model 2. For Model 2 K was underestimated.

New Estimation Approach to Reduce Estimation Bias

As there was a significant bias in B and K when using a MATLAB's nonlinear least-squares estimation algorithm, this bias had to be corrected. We focused on correcting the bias in B, as the K values were not central to the hypotheses of the study. We did not attempt to eliminate the σ^2 bias in Model 1, because Models 2 and 3 corrected this bias.

To improve the B estimates, we adopted the estimation approach used by Ahn and Hogan (2015) and Ahn et al. (2016). This method estimates B using an iterative learning model (described in Equation A1), substituting C for $(1-B)$:

$$x_{i+1} = Cx_i + N_{i+1} - N_i \qquad \text{(Equation A1)}$$

where x_i is the ith value in a time series, N_i is a random sample from a distribution with zero mean and standard deviation σ_N. Thus, N_{i+1}–N_i becomes a sample from a colored noise distribution. This trial-by-trial learning model has been used in human motor behavior analysis (van Beers 2009). The parameter C is between 0 and 1. Ahn et al. (2016) showed that estimation of this parameter from human data renders a significant bias, which can be corrected with an analytically derived adjustment, the Adjusted Yule-Walker (AYW) method (Yule 1927; Walker 1931).

The learning model in Equation A1 is a particular case of the learning model used in the present study with the noise ratio $K = 1$:

$$x_{i+1} = x_i\left(1-B\right) + N_{i+1} - KN_i \qquad \text{(Equation A2)}$$

It can be seen that Equation A2 is identical to Equation 15 in the main text. For convenience, we define:

$$C = 1 - B \qquad \text{(Equation A3)}$$

For both models, formulated for regression in Equations A1 and A2, the Yule-Walker-estimate of the parameter C is calculated as:

$$C_{YW} = \frac{\sum_{i=1}^{n-1} x_i x_{i+1}}{\sum_{i=1}^{n-1} x_i^2} \qquad \text{(Equation A4)}$$

Ahn et al. (2016) analytically derived the expression for Equation A4:

$$\sum_{i=1}^{n-1} x_i^2 = \left(\frac{\left(C-1\right)^2}{1-C^2} + 1\right)\left(n-1\right)\sigma_N^2 \qquad \text{(Equation A5)}$$

$$E\left[\sum_{i=1}^{n-1} x_i x_{i+1}\right] = (C-1)(n-1)\sigma_N{}^2 + (C-1)^2\frac{C}{1-C^2}\left\{(n-2) - \frac{C^2}{1-C^2}\left(1-C^{2(n-2)}\right)\right\}\sigma_N{}^2 \qquad \text{(Eq. A6)}$$

where C is the actual parameter value from the model and n is the length of the time series x_i, with $i = 1, \ldots\ n$. Note these expressions are for $K = 1$.

To extend this definition to include Model 2 with $K < 1$, the parameter K is inserted into Equation A5 and Equation A6 by replacing the term (C-1) with (C-K). The equations for the extended learning model (Equations A2 and A3) then become:

$$\sum_{i=1}^{n-1} x_i^{\,2} = \left(\frac{(C-K)^2}{1-C^2} + 1 \right)(n-1)\sigma_N^{\,2} \qquad \text{(Equation A7)}$$

$$E\left[\sum_{i=1}^{n-1} x_i x_{i+1} \right] = (C-K)(n-1)\sigma_N^{\,2} + (C-K)^2 \frac{C}{1-C^2} \left\{ (n-2) - \frac{C^2}{1-C^2}\left(1-C^{2(n-2)}\right) \right\} \sigma_N^{\,2} \qquad \text{(Eq. A8)}$$

Ahn et al. (2016) demonstrated that the bias induced by the YW method does not decrease when n is large. Inserting Equation A7 and Equation A8 into Equation A4, and taking the limit for $n \to \infty$, the expression for the expectation of C simplifies to:

$$E\left[C_{YW}\right] \cong \lim_{n\to\infty} \frac{E\left[\sum_{i=1}^{n-1} x_i x_{i+1} \right]}{\sum_{i=1}^{n-1} x_i^{\,2}} = \frac{(C-K)(1-KC)}{1+K^2-2KC} \qquad \text{(Equation A9)}$$

The subscript in C_{YW} denotes that the parameter was derived with the Yule-Walker method. The expectation of the bias can be calculated as:

$$E\left[Bias\right] = E\left[C_{YW}\right] - C \cong \frac{(C-K)(1-KC)}{1+K^2-2KC} - C = \frac{K\left(C^2-1\right)}{1+K^2-2KC} \qquad \text{(Equation A10)}$$

As Equation A10 does not contain trial length n or noise variance σ_N^2, the calculated bias is not sensitive to n or σ_N^2. The relation between C_{YW} and the actual C is:

$$\hat{C}_{YW} = C + Bias = C + \frac{K\left(C^2-1\right)}{1+K^2-2KC} \qquad \text{(Equation A11)}$$

After re-arranging and solving Equation A6 for the actual value C, the Adjusted Yule-Walker estimation of C_{AYW} is:

$$C_{AYW} = \hat{C} = \frac{\left(2K\hat{C}_{YW} + K^2 + 1\right) - \sqrt{\left(2K\hat{C}_{YW} + K^2 + 1\right)^2 - 4K\left(\hat{C}_{YW}\left(K^2+1\right) + K\right)}}{2K} \qquad \text{(Eq. A12)}$$

Therefore, the AYW-estimation of B in Equation A2 is:

$$B_{AYW} = 1 - C_{AYW} \qquad \text{(Equation A13)}$$

Validation of New Method for B Estimation for Models 1 and 2

Figure A1.2 displays the adjusted B-values, illustrating that the adjustment reduced the bias significantly. Note however, that calculations of larger B values were slightly less reliable.

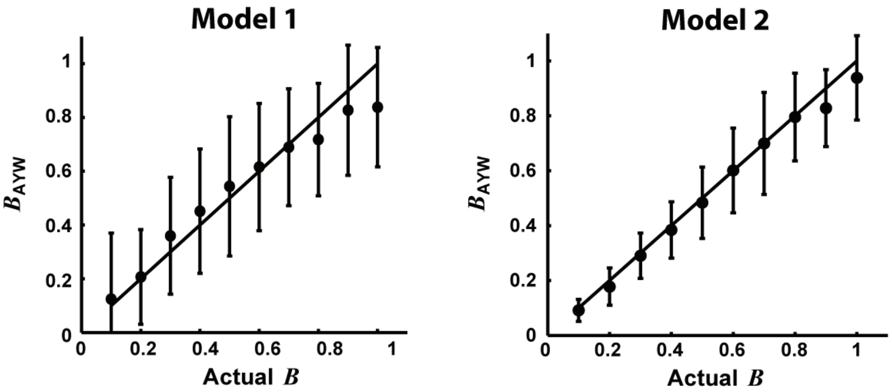

Figure A1.2. *Results of the system identification of the error correction gain B on Monte Carlo simulated time series for Models 1 and 2. The line shows the actual B-values, the data points represent the estimates of the Adjusted Yule-Walker method. The error bars represent one standard deviation across 100 simulations. For Model 1 σ^2=0.02; for Model 2 K=0.4 and σ^2=0.02.*

Validation of System Identification for Model 3

To validate the accuracy of parameter estimation of Model 3, Monte-Carlo simulations were performed for different parameter combinations of B, σ_{PL}^2, and σ_{EX}^2, again for 60 iterations each. 100 simulations were run for each parameter combination.

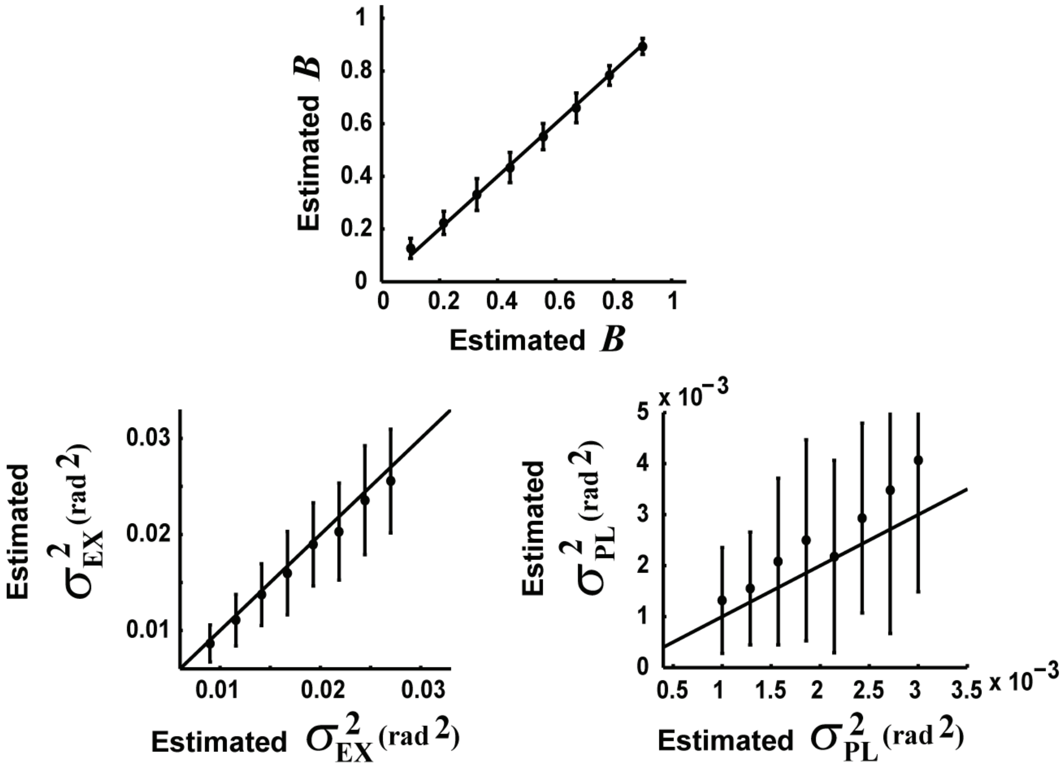

Figure A1.3. *Validation of Model 3 with two independent noise sources.*

References

Ahn, J., & Hogan, N. (2015). Improved assessment of orbital stability of rhythmic motion with noise. *PloS ONE*, 10(3), e0119596.

Ahn J, Zhang Z, Sternad D. (2016). Noise induces biased estimation of the correction gain. *PLoS ONE*, 11(7), e0158466.

van Beers RJ. Motor learning is optimally tuned to the properties of motor noise. *Neuron*. 2009;63(3):406-17.

Walker G. On periodicity in series of related terms. *Proc R Soc A*. 1931;131(818):518-32.

Yule GU. On a method of investigating periodicities in disturbed series, with special reference to Wolfer's sunspot numbers. *Phil Trans R Soc A*. 1927:267-98.
