# Supplementary material for: Neuromotor Noise Is Malleable by Amplifying Perceived Errors
Source: PLoS Comput Biol. 2016 Aug 4;12(8):e1005044. doi: 10.1371/journal.pcbi.1005044 (PMC4973920; doi:10.1371/journal.pcbi.1005044)
Supplement: S2 Appendix — (PDF) [file pcbi.1005044.s002.pdf]

Appendix 2

Results of System Identification for All Models

Error Correction Gain B: The estimated error correction gain B for Models 1 and 2 are shown in Figure A2.1 (using the unbiased estimate). Results of Model 3 are also reproduced from the main text to facilitate comparison. Model 1 tended to have the lowest B estimates, followed by Model 3. Model 2 had the largest B estimates. While the B values tended to increase with practice in the amplified conditions, the results of Models 1 and 2 show a relatively inconsistent pattern.

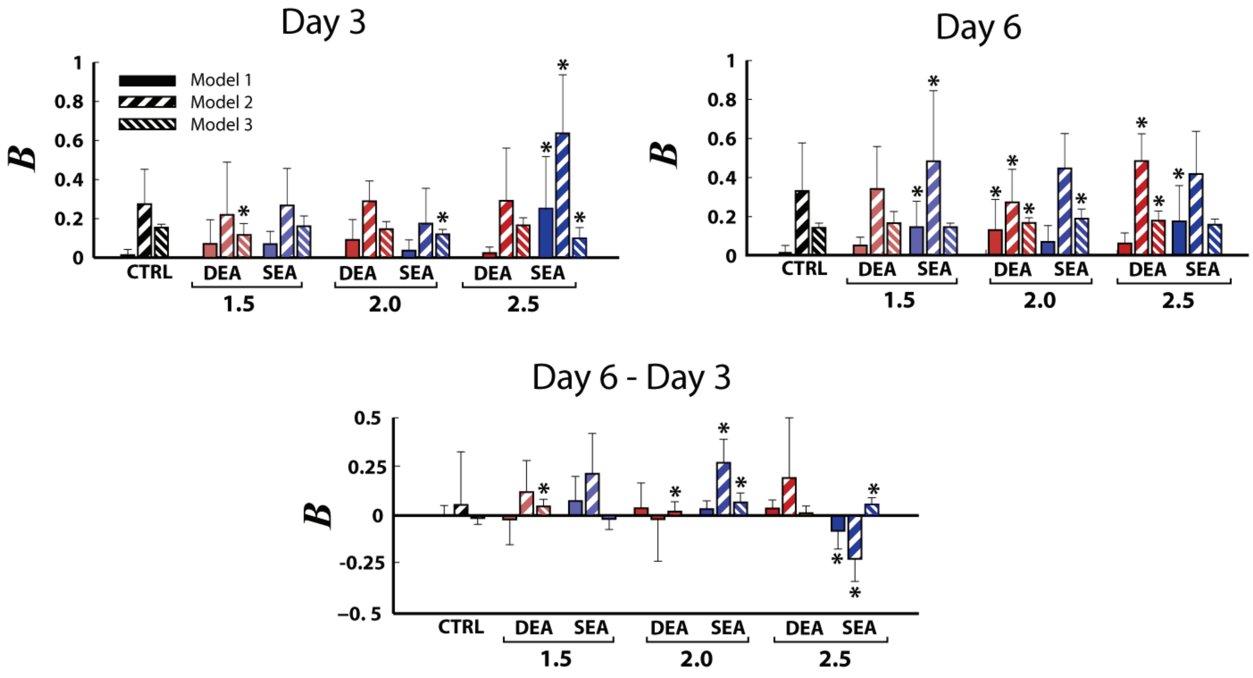

Figure A2.1. Estimates for the error correction gain B for all three models. *CTRL = control (no manipulation; black); DEA = deterministic error amplification (red), SEA = stochastic error amplification (blue). Three levels of amplification were used: 1.5, 2.0, and 2.5. Error bars show one between-subjects standard deviation. *Groups significantly different than the control group at p < 0.05.*

Noise Variance: Estimates for the noise variance are shown in Figure A2.2. To facilitate comparison across models, only the execution noise variance is shown for Model 3. Planning noise variance is not compared because the separate planning noise variance only existed for Model 3. All three models provided consistent results, showing that noise variance decreased with error amplification.

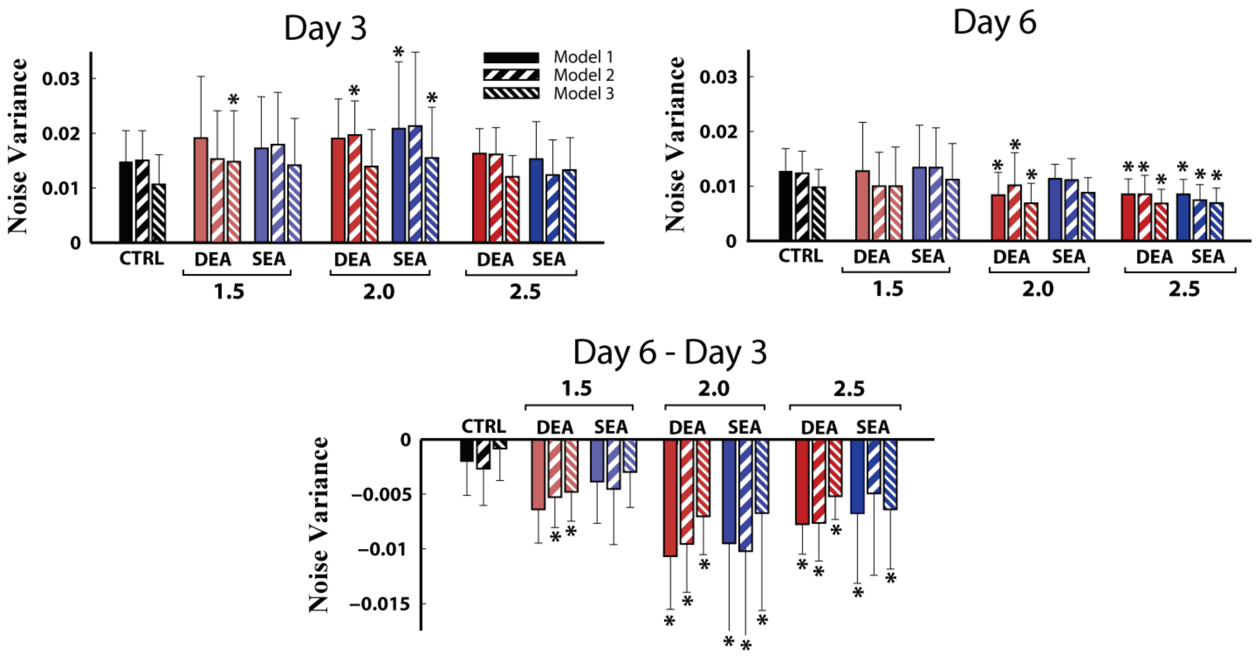

Figure A2.2. Estimates for the **noise variance** ***for the three models.*** *CTRL = control (no manipulation; black); DEA = deterministic error amplification (red), SEA = stochastic error amplification (blue). Three levels of amplification were used: 1.5, 2.0, and 2.5. Error bars show one between-subjects standard deviation. *Groups different than the control group at p < 0.05.*

Noise Ratio K: The estimated noise ratio K for Model 2 was negative in a significant number of analyzed blocks (383 out of 1008). These instances also had B-values larger than 1.0. As negative K-values were not compatible with the definition of K as a positive ratio of η_{EX} and η_{TOTAL} , these results indicated that the model was not appropriate for these particular blocks of trials/subjects. Restricting K to positive values was possible, but led to ceiling effects and distortions that we regarded as inappropriate. Such blocks were distributed across all subjects, conditions, and days. The estimates for K are shown in Figure A2.3. For Model 3, K was computed as:

$$K = \frac{{\sigma_{EX}}^2}{{\sigma_{PL}}^2 + {\sigma_{EX}}^2}$$

Only Models 2 and 3 are shown because $K = 1$ for Model 1. Only positive K values are included in the results for Model 2. The results show that in Model 3, K was consistently high; but in Model 2, K remained variable in mid-range.

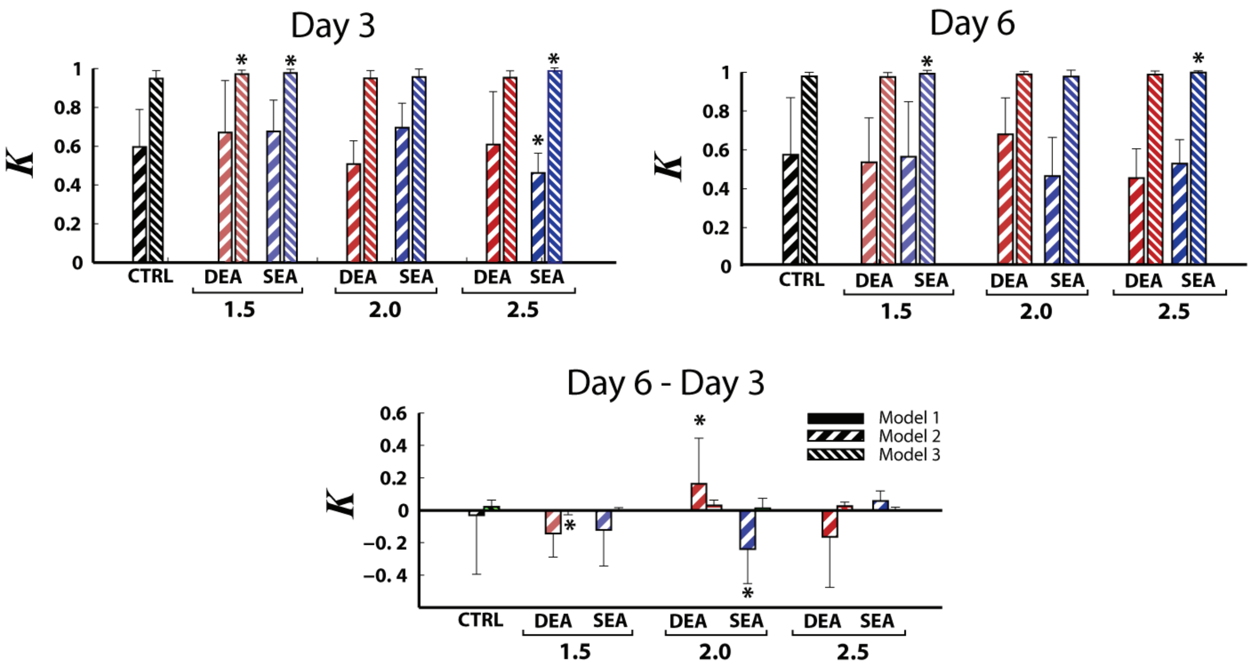

Figure A2.3. Estimates for the* noise ratio *K for Model 1 and 2. *CTRL = control with no manipulation (black); DEA = deterministic error amplification (red), SEA = stochastic error amplification (blue). Three levels of amplification were used: 1.5, 2.0, and 2.5. Error bars show one between-subjects standard deviation. *Groups significantly different than the control group at p < 0.05.*
